# Transcriptome Analysis and Identification of Chemosensory Membrane Proteins in the Head of *Euplatypus parallelus*

**DOI:** 10.3390/insects16050504

**Published:** 2025-05-07

**Authors:** Qi Wu, Xiang Zhou, Zheyuan Xu, Xufeng Zhang, Hongchao Yuan, Jixing Guo

**Affiliations:** Key Laboratory of Green Prevention and Control of Tropical Plant Diseases and Pests, Ministry of Education, Institute of Tropical Agriculture and Forestry, Hainan University, Haikou 570228, China; 22210904000001@hainanu.edu.cn (Q.W.); 991017@hainanu.edu.cn (X.Z.); 22220951320071@hainanu.edu.cn (Z.X.); 23210904000010@hainanu.edu.cn (X.Z.); 23210904000008@hainanu.edu.cn (H.Y.)

**Keywords:** chemosensory membrane protein, *Euplatypus parallelus*, RNA-seq, expression profile, bioinformatics analysis

## Abstract

*Euplatypus parallelus* is a polyphagous pest capable of harming multiple plant species. Adult beetles invade tree trunks by boring holes, which negatively impacts the trees’ growth and may result in tree death. *E. parallelus* depends on plant volatiles to identify and locate appropriate hosts for feeding or reproduction, with its olfactory system playing a vital role in volatile detection. In this work, we applied transcriptomics, phylogenetic analysis, and expression analysis to investigate four chemosensory membrane protein gene families that play a role in olfaction in *E. parallelus*. Based on the annotation analysis, 41 odorant receptors (ORs), 12 gustatory receptors (GRs), 14 ionotropic receptors (IRs), and 4 sensory neuron membrane proteins (SNMPs) were identified in the head. We used differential gene expression (DGE) and fragments per kilobase per million (FPKM) values to compare the transcription levels of chemosensory membrane protein gene families between males and females. The data indicate that the chemosensory membrane protein gene families in *E. parallelus* exhibit different expression levels in male and female heads, with some genes showing significant differences and displaying sex-biased expression. These results offer a basis for future exploration of the functions of chemosensory membrane protein gene families in *E. parallelus* and offer a theoretical framework for designing innovative eco-friendly control technologies.

## Simple Summary

*Euplatypus parallelus*, a significant pest recently discovered in China’s rubber planting areas, severely damages rubber trees. The adult beetles invade the trunks of rubber trees, drilling holes and affecting the healthy growth of the trees. Olfaction plays a fundamental role in how *E. parallelus* selects its hosts and is a key factor in the beetles’ ability to locate and drill into rubber tree trunks. We employed transcriptome sequencing and bioinformatics tools in this study to identify and analyze four types of chemosensory membrane proteins, covering essential chemosensory membrane protein gene families such as odorant receptors (ORs), ionotropic receptors (IRs), gustatory receptors (GRs), and sensory neuron membrane proteins (SNMPs). FPKM values were applied to examine transcriptional differences in chemosensory genes between males and females. This research provides molecular evidence supporting further exploration of chemosensory mechanisms governing host recognition, localization, and oviposition site selection in *E. parallelus*.

## 1. Introduction

The ambrosia beetle *Euplatypus parallelus* (Coleoptera, Curculionidae, Platypodinae) is a harmful and extensively spread invasive pest. Native to South America, it expanded its range through the timber trade in the late 19th century, and has since become widely established in Africa, Madagascar, Australia, Southeast Asia, and China [1,2,3,4]. It is now listed as a significant quarantine pest in the Catalogue of Quarantine Pests for Import Plants to the People’s Republic of China [3,5]. The beetle exhibits a broad host range, attacking multiple plant families, including Anacardiaceae, Fabaceae, Pinaceae, and Myrtaceae [6,7,8,9]. By boring through the phloem into the xylem, *E. parallelus* causes tree decline or mortality, compromises wood quality, and reduces economic value [7]. The invisibility and elusive behavior of *E. parallelus* pose significant challenges to chemical control, while the excessive use of pesticides raises serious environmental concerns. Utilizing semiochemicals, such as plant-derived volatiles and pheromones, has been verified as an effective biological strategy for managing this pest while reducing pesticide reliance.

Insects rely on their olfactory system for essential life functions, such as feeding, mating, oviposition, and locating host. These processes are crucial for their survival and reproductive success [10]. The antennae function as the primary sensory organs in insects for sensing external chemical cues. Odorant recognition in insects involves multiple olfactory-related proteins. Odorant binding proteins (OBPs) and chemosensory proteins (CSPs) represent two types of soluble olfactory proteins included, along with four types of chemosensory membrane proteins: ionotropic receptors (IRs), odorant receptors (ORs), gustatory receptors (GRs), and sensory neuron membrane proteins (SNMPs) [11,12]. Insect odorant receptors are distinguished by seven transmembrane domains. Unlike GPCRs in vertebrates, insect ORs exhibit an inverted membrane topology [13]. These receptors are integral to olfactory recognition, enabling insects to detect odor molecules and transmit olfactory signals to downstream neural circuits, which can trigger specific behaviors [14]. Odorant receptors are categorized into two groups: odorant receptors (ORs) and the odorant receptor co-receptor (ORco). OR and ORco proteins form tetrameric complexes that act as ion channels mediating olfactory signal transduction [15,16,17]. Recent research indicates that these complexes consist of OR and ORco subunits in a 1:3 ratio [18,19]. The membrane structures of GRs are similar to ORs, but their function includes detecting sugars, bitter compounds, and carbon dioxide [20]. IRs belong to the ionotropic glutamate receptor (iGluRs) subfamily, are capable of detecting amines and acids [21], and in some cases, they also participate in temperature and humidity sensing [22]. Insect sensory neuron membrane proteins (SNMPs) are membrane proteins located in sensory neurons [23]. Studies have indicated that SNMPs are particularly important for pheromone recognition [24]. Understanding the molecular mechanisms of insect chemosensory membrane proteins provides valuable insights into their olfactory system, offering potential avenues for regulating insect behavior.

The fast progress of high-throughput sequencing technologies in recent years has markedly expanded transcriptomic applications, including gene discovery, functional annotation, and genetic diversity analyses [25,26]. Numerous studies have identified and analyzed chemosensory protein genes in various insect species, such as *Sesamia inferens*, *Colaphellus bowringi*, *Calliphora stygia*, *Mythimna separata*, and *Sesamia nonagrioides* [27,28,29,30,31], providing a foundation for understanding chemosensory protein mechanisms in insects. However, little is known about the transcriptomic studies or the olfactory molecular mechanism of *E. parallelus*. In this study, high-throughput sequencing was employed to analyze the transcriptomes of male and female *E. parallelus* adult heads, and to identify and analyze chemosensory membrane protein genes. These results contribute to our understanding of the olfactory mechanisms in *E. parallelus* and offer a theoretical foundation for formulating sustainable and eco-friendly pest management strategies.

## 2. Materials and Methods

### 2.1. Insect Collection

*E. parallelus* specimens were collected from the area surrounding the Danzhou campus of Hainan University in Danzhou City, Hainan Province (19.30° N, 109.30° E). Healthy male and female adults (25 individuals of each sex) were selected, and their heads were removed, instantly frozen using liquid nitrogen and maintained at −80 °C.

### 2.2. RNA Extraction and cDNA Library Construction and Transcriptome Sequencing

RNA was extracted from the heads of three adult females (Epar-F1, Epar-F2, Epar-F3) and three adult males (Epar-M1, Epar-M2, Epar-M3). Total RNA from the heads of male and female *E. parallelus* was extracted using TRIzol reagent (Invitrogen, Carlsbad, CA, USA). The concentration and purity of total RNA were measured using a NanoDrop spectrophotometer (Thermo, Wilmington, DE, USA), and RNA integrity was assessed with an Agilent 2100 (Agilent Technologies, Santa Clara, CA, USA) to ensure the samples met the transcriptome sequencing standards. Once the concentration and purity of the total RNA from *E. parallelus* heads were confirmed, library construction was initiated. The process began by enriching eukaryotic mRNA using Oligo(dT)-coated magnetic beads, then randomly fragmenting the mRNA with fragmentation buffer. The first and second cDNA strands were synthesized using the mRNA as a template, and the cDNA was purified. Following purification, the double-stranded cDNA went through end repair, A-tailing, and adapter ligation. Size selection was performed using AMPure XP beads, and the cDNA library was enriched through PCR amplification. Upon completing the library construction, the initial quantification was performed using a Qubit 3.0 fluorometer (Waltham, MA, USA), with the required concentration ≥ 1 ng/µL. The insert fragments were then examined using the Qsep400 high-throughput analysis system to confirm the expected insert size, to ensure library quality, and Q-PCR was used to accurately measure the effective library concentration (effective concentration > 2 nM). Once the library passed quality control, sequencing was conducted on a high-throughput platform using the PE150 sequencing mode. The construction and sequencing of the cDNA library were performed by Beijing Biomarker Technologies Co., Ltd. (Beijing, China).

### 2.3. Transcriptome Assembly and Gene Annotation

Adapter-containing and low-quality reads were removed from the raw sequencing data (raw reads), such as those with more than 10% N bases or those with more than 50% bases having a quality score ≤ 10. Clean and high-quality data were successfully obtained following a series of quality control procedures. Trinity software (v2.14.0) was applied to split the sequencing reads into shorter fragments (K-mers), which were then extended into longer sequences (contigs). These fragments were assembled into component sets based on overlapping sequences. Using De Bruijn graph methods and sequencing read data, transcript sequences were identified within each component set. A high-quality unigene library was ultimately generated. DIAMOND software (v2.0.4) was used to align unigene sequences with NR, Swiss-Prot, COG, KOG, eggNOG4.5, and KEGG databases [32,33,34,35,36,37,38]. KEGG orthology results for unigene were obtained using KOBAS, and InterProScan was used to analyze the GO orthology of novel genes via the integrated InterPro database [39,40,41]. Following the prediction of unigene amino acid sequences, HMMER software (v3.1b2) was utilized to align them with the Pfam database for annotation purposes [42,43].

### 2.4. Gene Identification and Sequence Analysis

The head transcriptome data of *E. parallelus* were analyzed, and potential chemosensory protein genes were retrieved via NR annotation. The predicted gene sequences were subjected to sequence alignment using blastx (https://blast.ncbi.nlm.nih.gov/Blast.cgi, accessed on 2 September 2024), with alignment parameters including species name, gene description, accession number, E-value, and identity, leading to the identification of chemosensory proteins. The chemosensory protein genes in *E. parallelus* were examined for open reading frames (ORFs) using the ORF Finder online tool (https://www.ncbi.nlm.nih.gov/orffinder/, accessed on 2 September 2024). The odorant receptor gene sequences of *E. parallelus* were subjected to transmembrane domain prediction using the DeepTMHMM online tool (https://dtu.biolib.com/DeepTMHMM, accessed on 2 September 2024).

### 2.5. Phylogenetic Analysis of Chemosensory Membrane Protein

The chemosensory membrane protein genes (ORs, GRs, IRs, and SNMPs) identified in *E. parallelus* had duplicate sequences removed and a phylogenetic tree was constructed that includes chemosensory-related sequences from *E. parallelus* and other species such as the *Trypodendron lineatum*, *Ips typographus*, *Agrilus planipennis*, *Dendroctonus ponderosae*, and *Drosophila melanogaster*, to ensure the quality of the phylogenetic tree in our analysis, we primarily selected transmembrane chemosensory genes with more than 150 amino acids. We selected *T. lineatum*, *I. typographus*, and *D. ponderosae* primarily because they are phylogenetically close to *E. parallelus*, allowing for a more accurate analysis of their evolutionary relationships. Additionally, their annotated transmembrane chemosensory protein gene sets are relatively complete. *A. planipennis* and *D. melanogaster* were selected as outgroup species due to their distant phylogenetic relationships. *D. melanogaster* has been extensively studied in terms of GR and IR families, making it suitable for inferring evolutionary direction and rooting the phylogenetic tree. Multiple sequence alignment was performed using MAFFT (v7.520) [44], followed by gap region filtering using trimAl [45]. A phylogenetic tree of chemosensory protein genes was constructed using IQ-TREE software (v2.3.5) with 1000 bootstrap replicates, based on maximum likelihood estimation [46]. Figtree was employed for the visualization and beautification of the phylogenetic tree (http://tree.bio.ed.ac.uk/software/figtree, accessed on 5 September 2024). To examine the differences in the expression of chemosensory membrane protein genes in the heads of male and female *E. parallelus*, RSEM software (v1.2.19) was used for statistical analysis, and gene expression between males and females was quantified using FPKM values and expressed as heatmap [47].

## 3. Results

### 3.1. Transcriptome Sequencing and Assembly

We obtained a total of 41.57 Gb of high-quality reads, with each sample yielding more than 6.13 Gb of clean data. The Q30 base percentage exceeded 92.18%, and GC content ranged from 34.45% to 35.34% (Table 1). Transcript assembly using Trinity produced 58,362 transcripts with an N50 length of 2494 bp. Analysis of the assembled sequences yielded 32,775 unigenes, with an N50 length of 2104 bp, reflecting strong assembly integrity (Table 2).

### 3.2. Functional Annotation of the Unigenes in E. parallelus

The unigene sequences were aligned using Diamond software against eight major databases: NR, Swiss-Prot, GO, COG, KOG, eggNOG, Pfam, KEGG and STRING. The head transcriptome data of *E. parallelus* produced a total of 17,761 annotated unigenes, with the NR database providing the highest number (14,557 unigenes, 81.96%) (Table 3).

### 3.3. Identification and Analysis of Candidate Chemosensory Membrane Protein Genes

#### 3.3.1. The OR Gene Family

**Figure 1:**
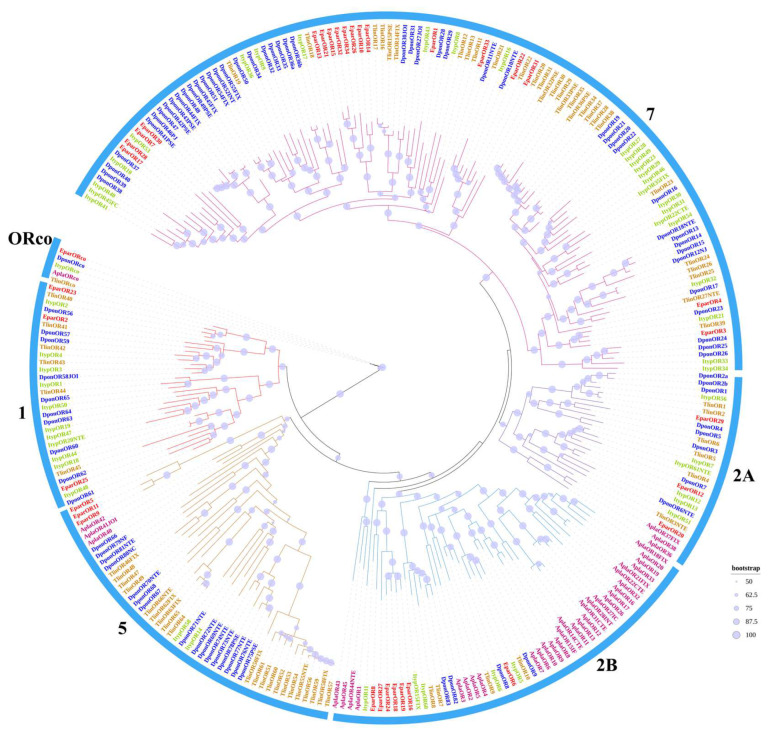
Phylogenetic tree of odorant receptors in *E. parallelus* and other insects. Phylogenetic trees were constructed using ORs sequences from *E. parallelus* and selected Coleoptera species, with ORco as the basal node. Different colors were used to highlight each Coleoptera OR subfamily, and respective numbers were annotated. The OR color for *E. parallelus* (Epar) is red, while the following colors are used for other species: Tlin, *T. lineatum* (orange); Ityp, *I. typographus* (green); Apla, *A. planipennis* (deep pink); Dpon, *D. ponderosae* (blue). The size of the dots reflects their bootstrap values. The larger dots indicate larger bootstrap values.

A total of 41 OR genes were predicted from the transcriptome of *E. parallelus,* with the highly conserved co-receptor named EparORco, and the other 40 genes named EparOR1-EparOR40. Among the 41 EparORs, 19 genes have complete open reading frames (ORFs), encoding proteins of 378 to 483 amino acids with 6–7 TMDs, which is characteristic of insect ORs [48]. The remaining 22 genes (EparOR19-EparOR40) have ORFs encoding proteins ranging from 45 to 363 amino acid residues, identified as fragment gene sequences (Table 4 and Supplementary Table S1). The entire protein sequences of *E. parallelus* EparORs are provided in Supplementary Text S1. To investigate the phylogeny of *E. parallelus* ORs and other Coleoptera species, the phylogenetic tree was built using the 34 OR sequences identified in *E. parallelus* and 242 OR sequences from four other insect species (Figure 1). The phylogenetic tree reveals that the ORs of these five Coleoptera species can be divided into six major clades, with EparORco clustering with other Coleoptera ORcos in one clade, and the 34 EparORs distributed across six branches of the evolutionary tree, mainly in subfamilies 2B and 7 [49,50,51].

#### 3.3.2. The GR Gene Family

**Figure 2:**
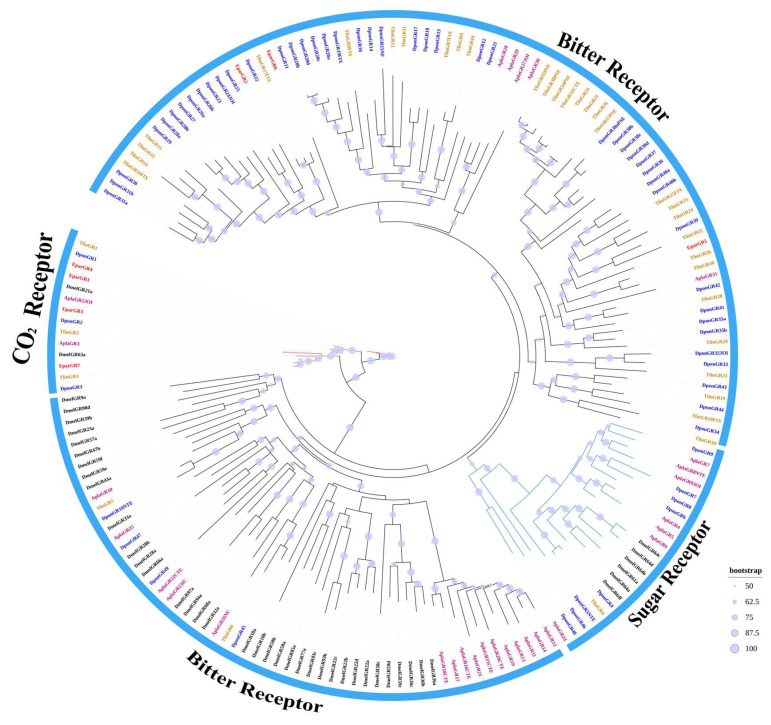
Phylogenetic tree of gustatory receptors in *E. parallelus* and other insects. A phylogenetic tree was constructed using the GR sequences of *E. parallelus* and other species, with each species highlighted in different colors. The GRs of *E. parallelus* (Epar) are shown in red, while the colors for other species are as follows: Dmel, *D. melanogaster* (black); Apla, *A. planipennis* (deep pink); Dpon, *D. ponderosae* (blue); Tlin, *T. lineatum* (orange). The size of the dots reflects their bootstrap values. The larger dots indicate larger bootstrap values.

From the transcriptome of *E. parallelus*, 12 GRs were identified and named EparGR1-EparGR12. Four of these have complete open reading frames (ORFs), encoding proteins with 400 to 444 amino acids, while the other 8 EparGRs have incomplete gene sequences (Table 5 and Supplementary Table S1). The entire protein sequences of *E. parallelus* EparGRs are provided in Supplementary Text S1. The phylogenetic tree was built using 7 identified EparGR sequences and 170 GR sequences from four other insect species (Figure 2). The phylogenetic analysis shows that four GRs cluster with carbon dioxide receptors, while three GRs cluster with bitter receptors.

#### 3.3.3. The IR and iGluR Gene Family

**Figure 3:**
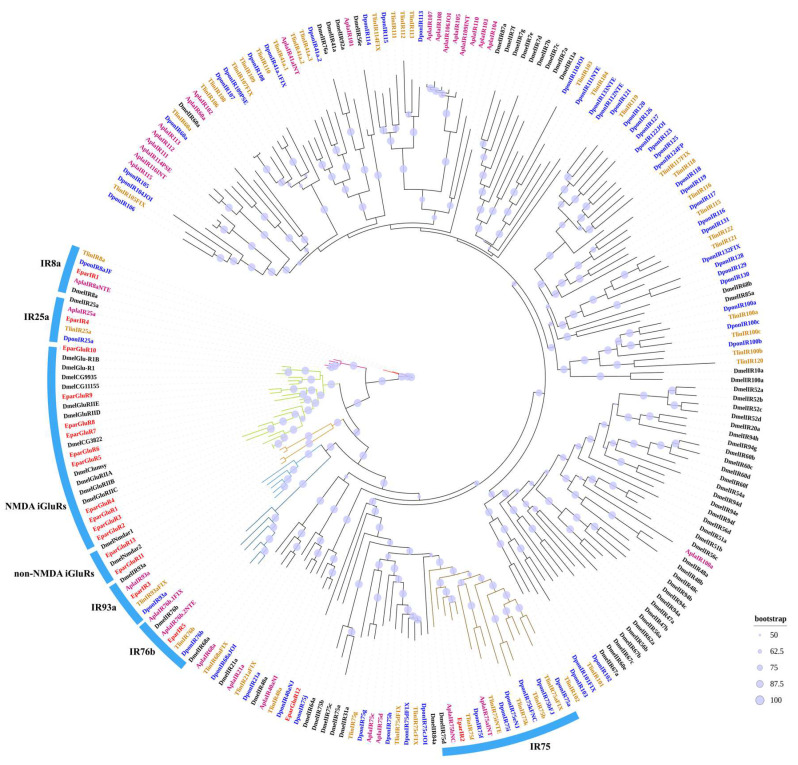
Phylogenetic tree of ionotropic receptors and ionotropic glutamate receptors in *E. parallelus* and other insects. A phylogenetic tree was constructed using the IR and iGluR sequences of *E. parallelus* and other species, with each species highlighted in different colors. The IRs and iGluRs of *E. parallelus* (Epar) are shown in red, while the colors for other species are as follows: Dpon, *D. ponderosae* (blue); Dmel, *D. melanogaster* (black); Tlin, *T. lineatum* (orange); Apla, *A. planipennis* (deep pink). The size of the dots reflects their bootstrap values. The larger dots indicate larger bootstrap values.

In the transcriptome, a total of 14 IR-encoding transcripts were identified, with five of the IR sequences possessing complete ORFs, ranging from 478 to 925 amino acid residues and containing three TMDs. The other IR sequences are incomplete, with 5′ or 3′ ends missing (Table 6 and Supplementary Table S1). These IRs were named EparIR1-EparIR14. Additionally, 15 iGluR genes were identified, and the iGluR sequences were named EparGluR1-EparGluR15. Among these, 13 have complete ORFs, encoding amino acids ranging from 674 to 1058, while the remaining 4 sequences are incomplete (Table 6 and Supplementary Table S1). The entire protein sequences of both EparIRs and EparGluRs from *E. parallelus* are provided in Supplementary Text S1. The phylogenetic tree was built using 5 identified EparIR sequences, 12 EparGluR sequences, and 212 IR and iGluR sequences from four other insect species (Figure 3). The phylogenetic tree showed that IR1 clustered with IR8a, IR4 clustered with IR25a, IR5 clustered with IR76b, IR3 clustered with IR93a, and IR2 clustered with IR75s. EparGluR1-EparGluR10 grouped with NMDA iGluRs in one major clade, while EparGluR11-EparGluR13 grouped with non-NMDA iGluRs in another major clade.

#### 3.3.4. The SNMP Gene Family

**Figure 4:**
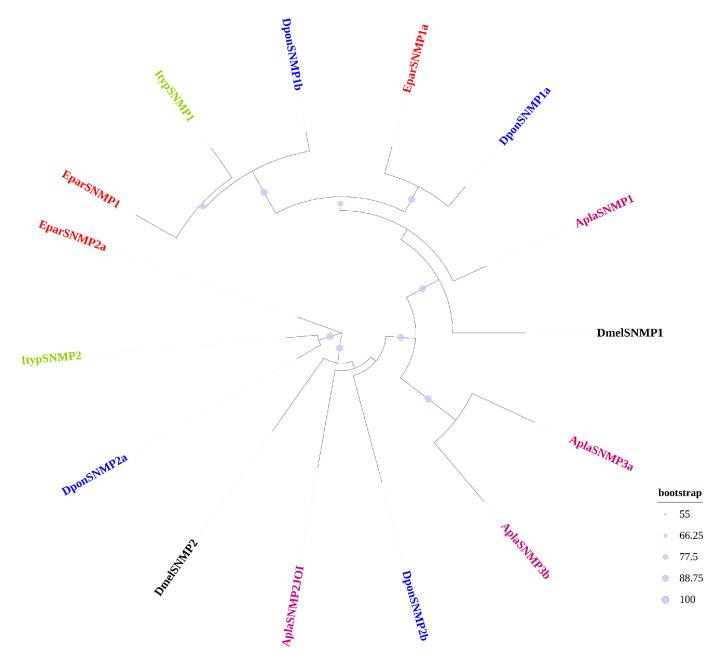
Phylogenetic tree of sensory neuron membrane proteins in *E. parallelus* and other insects. A phylogenetic tree was constructed using the SNMP sequences of *E. parallelus* and other species, with each species highlighted in different colors. The SNMP of *E. parallelus* (Epar) are shown in red, while the colors for other species are as follows: Dpon, *D. ponderosae* (blue); Ityp, *I. typographus* (green); Tlin, *T. lineatum* (orange); Apla, *A. planipennis* (deep pink); Dmel, *D. melanogaster* (black). The size of the dots reflects their bootstrap values. The larger dots indicate larger bootstrap values.

Four SNMPs were identified in the transcriptome of *E. parallelus*, with three being full-length sequences, and their amino acid residues range from 508 to 538, while one sequence is incomplete (Table 7 and Supplementary Table S1). The entire protein sequences of *E. parallelus* EparSNMPs are provided in Supplementary Text S1. These four EparSNMPs were named according to the Coleoptera homologous nomenclature system [52]. The phylogenetic tree was built using three identified EparSNMP sequences and 12 SNMP sequences from five other insect species (Figure 4). The results from the phylogenetic tree demonstrated that the three SNMPs were located in different branches and could be classified as EparSNMP1, EparSNMP1a, and EparSNMP2a.

#### 3.3.5. Expression Profiles of Chemosensory Membrane Protein Genes Between Sexes

**Figure 5:**
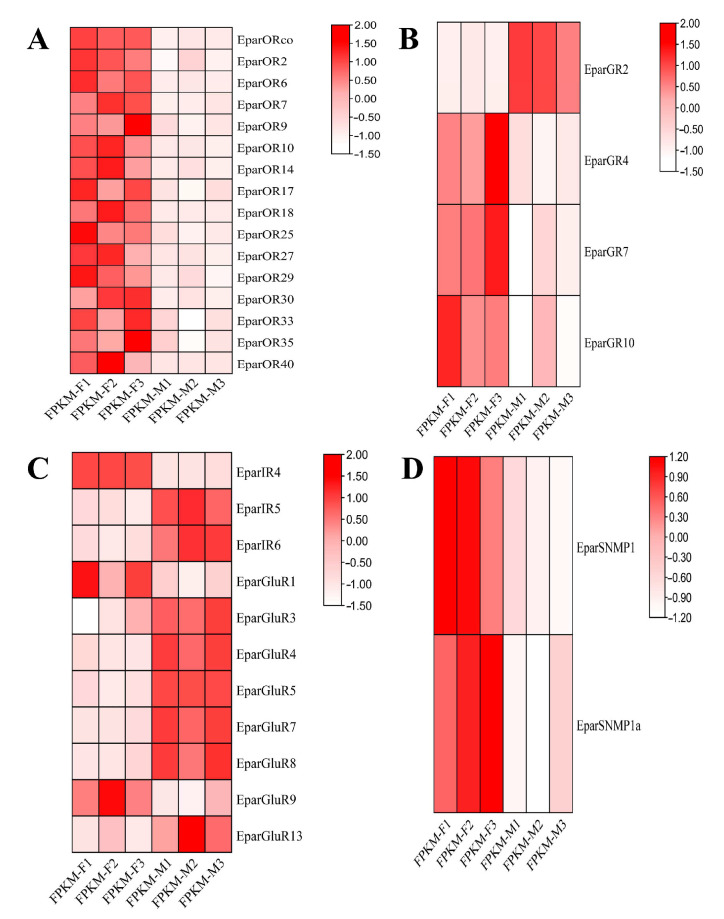
The heatmap displays the expression changes in chemosensory genes in *E. parallelus*, where colors represent the transcript abundance in transcript per million (Log2(FPKM+1)), with red indicating the highest levels and blue the lowest. (**A**) Odorant receptors (ORs), (**B**) gustatory receptors (GRs), (**C**) ionotropic receptors (IRs), (**D**) sensory neuron membrane proteins (SNMPs). Significant differences between males and females are marked by an asterisk at the 0.05 level.

Differential gene expression analysis revealed that chemosensory membrane protein expression varied between male and female heads (Figure 5 and Supplementary Table S2). Among the ORs, sixteen receptors, such as EparORco, EparOR2, EparOR6, EparOR7, EparOR9, EparOR10, EparOR12, EparOR14, and EparOR17, showed significant differences in expression levels between male and female individuals, and EparORco has the highest FPKM value of 63.43 in females. EparOR4 showed no significant differences, but expression levels exceeded 20 in both sexes. Among GRs, significant differences in expression were found in EparGR2, EparGR4, EparGR7 and EparGR10. Across male and female heads, EparGR10 exhibited significantly higher expression in males compared to females. In IRs and iGluRs, Significant expression differences in 11 genes were observed between the heads of males and females, including EparIR4, EparIR5, EparIR6, EparGluR1, EparGluR3, EparGluR4, EparGluR5, EparGluR7, EparGluR8, EparGluR9, and EparGluR13, EparGluR8 has the highest FPKM value of 625.43 in males. Among SNMPs, two genes exhibited significant differences, with expression levels being significantly higher in females than in males.

## 4. Discussion

*E. parallelus* is the major pest, posing a significant threat to the natural rubber tree industry. Chemosensory membrane protein genes, including odorant receptors (ORs), gustatory receptors (GRs), ionotropic receptors (IRs), and sensory neuron membrane proteins (SNMPs), are essential components of the olfactory system, responsible for the detection and perception of chemical odors [53]. This study involved performing transcriptome sequencing on *E. parallelus*, identifying a total of 86 chemosensory membrane protein genes (41 ORs, 12 GRs, 29 iGluRs, and 4 SNMPs). This is the first report of chemosensory gene identification in a species of the subfamily Platypodinae. Previous studies have identified chemosensory membrane genes in several Curculionidae beetles: *D. ponderosae* (207 genes), *Hypothenemus hampei* (162 genes), and *Trypodendron lineatum* (149 genes) through genome sequencing; and *Ips duplicatus* (150 genes), *Ips typographus* (89 genes), *Tomicus yunnanensis* (23 genes), and *Rhynchophorus ferrugineus* (108 genes) based on transcriptome sequencing [12,50,54,55,56,57]. The number of chemosensory membrane protein genes varies among these insect species, possibly reflecting differences in their biology or ecological adaptations [50,58,59,60]. Comparative analyses on wood-boring beetles revealed that host-specific species, such as the Pinus specialist *D. ponderosae*, the Fraxinus specialist *Agrilus planipennis*, and the ambrosia beetle *T. lineatum*, possess fewer chemosensory membrane protein genes compared to polyphagous species like *Tribolium castaneum* and *Anoplophora glabripennis*. This suggests that host-specific beetles may generally exhibit a reduced number of chemosensory membrane protein genes compared to those with broader host ranges [50,58,59,60]. *E. parallelus* is considered a polyphagous beetle, exhibiting a broad host range across multiple plant families, including Anacardiaceae, Fabaceae, Pinaceae, and Myrtaceae [6,7,8,9]. However, our transcriptome data did not reveal an expanded number of chemosensory membrane protein genes expressed in the head. In fact, compared with other species studied by transcriptome sequencing, the number of identified chemosensory membrane protein genes was relatively low. Differences in sampled tissues (head rather than isolated antennae) and sequencing depth may partially explain this discrepancy. Additionally, as an ambrosia beetle, the specialized ecological niche of *E. parallelus* might also contribute to its reduced number of chemosensory membrane protein genes. Most ambrosia and bark beetles maintain symbiotic relationships with microbes, particularly filamentous fungi, which help neutralize conifer chemical defenses or function as key nutritional resources [51,61,62,63]. Among ambrosia beetles, only *T. lineatum* has a fully annotated set of chemoreceptor genes derived from genomic data [60]. The diet of bark beetles like *D. ponderosae* and *I. typographus* includes fungi and the chemically defended phloem of their host trees. In contrast, *T. lineatum* exclusively feeds on its symbiotic fungus, *Phialophoropsis ferruginea*. Moreover, *T. lineatum* utilizes only a single-component aggregation pheromone and shows no responses to common scolytine pheromone compounds, reducing its need for a large number of pheromone receptors. However, for *E. parallelus*, it remains unclear whether it exclusively feeds on fungi or what pheromone components it employs. The relatively low number of chemosensory membrane protein genes identified in this study may provide important clues regarding its specialized ecological adaptations. Further genomic-level studies or increased RNA sampling specifically from *E. parallelus* antennae are needed to uncover additional chemosensory membrane protein genes.

Odorant receptors (ORs) serve a critical function in odor recognition and serve as the molecular basis for olfactory perception [64]. In this study, a total of 41 ORs were identified. Phylogenetic analysis revealed that EparORs are dispersed throughout the phylogenetic tree, with some clustering in specific subfamilies, particularly 2B and 7. These two subfamilies of odorant receptors have been previously reported to be enriched in certain bark and ambrosia beetles, where they are implicated in host and mate recognition [65,66]. The enrichment of EparORs within these groups suggests their potential role in detecting critical environmental cues, such as plant volatiles and pheromonal compounds. Phylogenetic analysis revealed that EparOR6 is closely grouped with ItypOR5 from *Ips typographus*, DponOR9 from *Dendroctonus ponderosae*, and TlinOR10 from *Trypodendron lineatum*. Previous studies have demonstrated that ItypOR5 exhibits highly specific responses to green leaf volatile (GLV) alcohols derived from angiosperms, including (E)-2-hexenol, (Z)-2-hexenol, (Z)-3-hexenol, and 1-hexanol [65]. Notably, DponOR9 has been confirmed to function similarly to ItypOR5 [65], suggesting that these orthologous genes exhibit functional conservation across different species and play identical roles in recognizing the same compounds. Given its strong phylogenetic relationship with ItypOR5 and DponOR9, EparOR6 is likely to perform a similar function in *E. parallelus* by detecting GLV alcohols, making it a promising target for further investigation. GLVs, which are abundant in the leaves of angiosperms and less so in conifers, may serve as an avoidance mechanism for *I. typographus* and *D. ponderosae*, preventing them from colonizing and reproducing on angiosperms [67]. However, unlike these conifer-specialized beetles, *E. parallelus* exhibits a broad host range, and GLVs are widely present in the volatile profiles of its host plants, including rubber trees [1]. This suggests that, despite sharing similar ligand recognition capabilities at the molecular level, their ecological roles and behavioral consequences may differ. Given the widespread presence of GLVs in *E. parallelus* host plants, EparOR6 may play a crucial role in host plant recognition. Furthermore, 1-hexanol has also been identified as a component of male-emitted volatiles. While no studies have specifically reported whether 1-hexanol alone functions as an attractant for conspecifics, male volatile blends have been shown to significantly enhance female attraction [68]. This suggests that 1-hexanol may be a potential pheromone component. Expression pattern analysis revealed that EparOR6 is specifically expressed in the female head, suggesting a possible role in pheromone detection. Studies have shown that HparOR27 is antenna-biased expressed in female *Holotrichia parallela* and can specifically recognize hexanal, lauric acid, and tetradecane [69]. MmedOR48 is biasedly expressed in female *Microplitis mediator* and can detect various plant volatiles [70]. Additionally, BmOR19, BmOR30, BmOR45, and BmOR47 are biasedly expressed in female *Bombyx mori* and are involved in detecting male pheromones [71]. In this study, nine ORs (including ORco) were identified as being female-biased expressed in the head of *E. parallelus*, suggesting their potential role in host plant localization or male pheromone recognition.

Gustatory receptors can recognize non-volatile compounds, including sugars, bitter compounds, and the gas carbon dioxide [72]. This study identified a total of 12 GRs in *E. parallelus*. Phylogenetic analysis classified the EparGRs into various clades, each with a specific predicted function. Four EparGRs were classified as carbon dioxide receptors, specifically GR1, GR3, GR4, and GR7, while three EparGRs were identified as bitter receptors: GR2, GR5, and GR6. The absence of sugar receptors may be due to the low expression levels of sugar receptor genes in the insect head.

The ionotropic receptor IR, along with its co-receptors IR8a, IR25a, and IR76b, functions as a ligand receptor [22]. In *E. parallelus*, we identified 29 proteins from the ionotropic glutamate receptor (iGluR) family. Based on sequence characteristics and phylogenetic analysis, they were divided into the iGluRs family and the IR family. Phylogenetic analysis revealed that EparIRs clustered with IR8a, IR25a, IR75s, IR76b, and IR93a from other species. This suggests they may have similar functions.

SNMP plays a potential role in pheromone detection and may also be involved in the pheromone clearance process [73,74]. In this study, four SNMPs were identified in *E. parallelus*, and the classification separates them into two groups: SNMP1 and SNMP2. SNMP1 may be involved in pheromone detection, while SNMP2 may play a role in pheromone clearance. The female head shows a significant increase in the expression of three SNMPs compared to the male head, suggesting their role in female recognition of male pheromones.

## 5. Conclusions

In summary, using high-throughput sequencing technology integrated with bioinformatics, this study conducted transcriptome sequencing and analysis of the head tissues of *E. parallelus*. A relatively complete transcriptome dataset for male and female adults was obtained, a total of 86 chemosensory membrane protein genes were successfully screened, including 41 ORs, 12 GRs, 14 IRs, 15 iGluRs, and 4 SNMPs. Their sequences, phylogenetic relationships, and relative expression levels were systematically analyzed. These findings lay the groundwork for functional validation of chemosensory genes in *E. parallelus* and provide a theoretical basis for designing innovative and environmentally sustainable pest control strategies.

## Figures and Tables

**Table 1:** The results of transcriptome sequencing from the head of *E. parallelus*.

Sample	Read Number	Base Number	GC Content	% ≥ Q30
Epar-F-1	21,805,037	6,525,067,675	35.34%	92.18%
Epar-F-2	22,408,048	6,693,904,718	34.73%	92.74%
Epar-F-3	24,058,796	7,197,880,307	35.34%	94.02%
Epar-M-1	25,618,478	7,661,751,802	34.45%	92.94%
Epar-M-2	20,465,126	6,125,589,791	35.11%	92.78%
Epar-M-3	24,621,535	7,367,886,987	34.75%	92.92%

**Table 2:** The results of transcriptome assembly results of *E. parallelus*.

Length Range	Transcript	Unigene
200–300	11,098 (19.02%)	9699 (29.59%)
300–500	8685 (14.88%)	6056 (18.48%)
500–1000	11,779 (20.18%)	6454 (19.69%)
1000–2000	12,723 (21.80%)	5341 (16.30%)
2000+	14,077 (24.12%)	5225 (15.94%)
Total Number	58,362	32,775
Total Length	83,550,564	35,529,222
N50 Length	2494	2104
Mean Length	1431.59	1084.03

**Table 3:** Annotation results of gene function in the head transcriptome of *E. parallelus*.

Database	Number of Annotated Unigenes	300 ≤ Length < 1000	Length ≥ 1000
COG_Annotation	4608	1180	3009
GO_Annotation	14,295	4202	8068
KEGG_Annotation	11,014	2742	7259
KOG_Annotation	10,113	2575	6536
Pfam_Annotation	12,919	3566	8037
Swissprot_Annotation	7769	1803	5365
eggNOG_Annotation	11,575	3040	7515
nr_Annotation	14,557	4152	8808
All_Annotated	17,761	5658	9296

**Table 4:** Best BLASTX matches of *E. parallelus* ORs.

Gene Name	Best Blast Mach	Gene Description	Acc. Number	E-Value	Identity (%)
*EparORco*	*Pachyrhinus yasumatsui*	odorant receptor co-receptor	UUW42911.1	0.00	86.1
*EparOR1*	*Eucryptorrhynchus scrobiculatus*	odorant receptor 13	QXE93194.1	4.00 × 10^−81^	42.75
*EparOR2*	*Pachyrhinus yasumatsui*	odorant receptor 19	WJJ63319.1	1.00 × 10^−147^	57.07
*EparOR3*	*Eucryptorrhynchus scrobiculatus*	odorant receptor 19	QXE93200.1	2.00 × 10^−137^	52.84
*EparOR4*	*Pachyrhinus yasumatsui*	odorant receptor 10	WJJ63310.1	8.00 × 10^−127^	50
*EparOR5*	*Anthonomus grandis grandis*	gustatory and odorant receptor 22-like	XP_050295302.1	0.00	80.09
*EparOR6*	*Eucryptorrhynchus scrobiculatus*	odorant receptor 3	QXE93184.1	3.00 × 10^−155^	52.47
*EparOR7*	*Pachyrhinus yasumatsui*	odorant receptor 7	WJJ63307.1	1.00 × 10^−76^	36.48
*EparOR8*	*Pachyrhinus yasumatsui*	odorant receptor 4	WJJ63304.1	1.00 × 10^−86^	38.38
*EparOR9*	*Euwallacea similis*	gustatory and odorant receptor 22-like isoform X1	KAF7267635.1	0.00	73.95
*EparOR10*	*Euwallacea similis*	odorant receptor 10-like	XP_066258781.1	1.00 × 10^−31^	29.32
*EparOR11*	*Sitophilus oryzae*	gustatory and odorant receptor 22	XP_030746043.1	0.00	74.81
*EparOR12*	*Eucryptorrhynchus scrobiculatus*	odorant receptor 1	QXE93182.1	5.00 × 10^−115^	49.34
*EparOR13*	*Pachyrhinus yasumatsui*	odorant receptor 24	WJJ63324.1	2.00 × 10^−80^	36.81
*EparOR14*	*Cylas formicarius*	odorant receptor 49b-like isoform X2	XP_060524832.1	3.00 × 10^−24^	32.01
*EparOR15*	*Pachyrhinus yasumatsui*	odorant receptor 26	WJJ63326.1	5.00 × 10^−31^	28.28
*EparOR16*	*Cylas formicarius*	odorant receptor 49b-like isoform X3	XP_060520304.1	2.00 × 10^−55^	32.5
*EparOR17*	*Dendroctonus ponderosae*	odorant receptor 47b	XP_019753281.2	2.00 × 10^−79^	39.9
*EparOR18*	*Cylas formicarius*	odorant receptor 49b-like isoform X3	XP_060520304.1	6.00 × 10^−62^	34.58
*EparOR19*	*Rhynchophorus ferrugineus*	odorant receptor	QCS37751.1	4.00 × 10^−24^	31.52
*EparOR20*	*Sitophilus oryzae*	odorant receptor 94a-like	XP_030753544.1	5.00 × 10^−99^	43.82
*EparOR21*	*Sitophilus oryzae*	odorant receptor 49b-like	XP_030764608.1	1.00 × 10^−72^	37.42
*EparOR22*	*Sitophilus oryzae*	odorant receptor 49b-like	XP_030764608.1	8.00 × 10^−116^	61.7
*EparOR23*	*Sitophilus oryzae*	odorant receptor 67c-like	XP_030758890.1	3.00 × 10^−71^	56.25
*EparOR24*	*Cylas formicarius*	odorant receptor 4-like isoform X2	XP_060520303.1	8.00 × 10^−56^	34.51
*EparOR25*	*Pachyrhinus yasumatsui*	odorant receptor 1	WJJ63301.1	9.00 × 10^−75^	50.92
*EparOR26*	*Cylas formicarius*	odorant receptor 45b-like isoform X1	XP_060530731.1	6.00 × 10^−32^	29.54
*EparOR27*	*Sitophilus oryzae*	odorant receptor 46a-like	XP_030763857.1	3.00 × 10^−30^	37.64
*EparOR28*	*Sitophilus oryzae*	odorant receptor 67c-like	XP_030750008.1	6.00 × 10^−63^	46.03
*EparOR29*	*Eucryptorrhynchus scrobiculatus*	odorant receptor 5	QXE93186.1	5.00 × 10^−78^	38.94
*EparOR30*	*Pachyrhinus yasumatsui*	odorant receptor 7	WJJ63307.1	9.00 × 10^−59^	36.84
*EparOR31*	*Eucryptorrhynchus brandti*	odorant receptor 26	QXE93252.1	5.00 × 10^−72^	41.95
*EparOR32*	*Pachyrhinus yasumatsui*	odorant receptor 26	WJJ63326.1	2.00 × 10^−28^	33.78
*EparOR33*	*Eucryptorrhynchus scrobiculatus*	odorant receptor 29	QXE93210.1	5.00 × 10^−33^	38.97
*EparOR34*	*Pachyrhinus yasumatsui*	odorant receptor 26	WJJ63326.1	2.00 × 10^−36^	33.61
*EparOR35*	*Pachyrhinus yasumatsui*	odorant receptor 26	WJJ63326.1	1.00 × 10^−36^	35.78
*EparOR36*	*Sitophilus oryzae*	odorant receptor 67a-like	XP_030763199.1	1.00 × 10^−12^	62
*EparOR37*	*Ips typographus*	odorant receptor 40	WZI48996.1	7.00 × 10^−19^	39.29
*EparOR38*	*Sitophilus oryzae*	odorant receptor 46a-like	XP_030763857.1	4.00 × 10^−09^	33.96
*EparOR39*	*Sitophilus oryzae*	odorant receptor 67a-like	XP_030763199.1	1.00 × 10^−12^	62
*EparOR40*	*Cylas formicarius*	odorant receptor Or2-like	XP_060533836.1	3.00 × 10^−12^	48.15

**Table 5:** Best BLASTX matches of *E. parallelus* GRs.

Gene Name	Best Blast Mach	Gene Description	Acc. Number	E-Value	Identity (%)
*EparGR1*	*Sitophilus oryzae*	gustatory and odorant receptor 22	XP_030746043.1	0	74.81
*EparGR2*	*Pyrrhalta aenescens*	gustatory receptor 13	APC94340.1	5 × 10^−7^	44.32
*EparGR3*	*Anthonomus grandis grandis*	gustatory and odorant receptor 22-like	XP_050295302.1	0	80.09
*EparGR4*	*Pachyrhinus yasumatsui*	gustatory receptor 1	WJJ63341.1	0	73.79
*EparGR5*	*Pachyrhinus yasumatsui*	gustatory receptor 9	WJJ63349.1	9 × 10^−7^	32.81
*EparGR6*	*Sitophilus oryzae*	gustatory receptor family protein 3-like	XP_030759073.1	9 × 10^−63^	68.16
*EparGR7*	*Anthonomus grandis grandis*	gustatory and odorant receptor 24	XP_050306870.1	1 × 10^−135^	85.98
*EparGR8*	*Colaphellus bowringi*	gustatory receptor 1	ALR72527.1	9 × 10^−16^	54.41
*EparGR9*	*Sitophilus oryzae*	gustatory receptor for sugar taste 64a-like	XP_030761257.1	3 × 10^−31^	41.71
*EparGR10*	*Colaphellus bowringi*	gustatory receptor 1	ALR72527.1	3 × 10^−11^	63.83
*EparGR11*	*Anthonomus grandis grandis*	gustatory receptor for sugar taste 43a-like isoform X1	XP_050302234.1	1 × 10^−39^	61.64
*EparGR12*	*Pachyrhinus yasumatsui*	gustatory receptor 6	WJJ63346.1	2 × 10^−19^	40.32

**Table 6:** Best BLASTX matches of *E. parallelus* IRs and iGluRs.

Gene Name	Best Blast Mach	Gene Description	Acc. Number	E-Value	Identity (%)
*EparIR1*	*Pachyrhinus yasumatsui*	ionotropic receptor 8a	WJJ63357.1	0	68.01
*EparIR2*	*Pachyrhinus yasumatsui*	ionotropic receptor 75s	WJJ63351.1	0	64.11
*EparIR3*	*Pachyrhinus yasumatsui*	ionotropic receptor 93a	WJJ63358.1	0	69.4
*EparIR4*	*Sitophilus oryzae*	ionotropic receptor 25a	XP_030756779.1	0	84.33
*EparIR5*	*Pachyrhinus yasumatsui*	ionotropic receptor 76b	WJJ63350.1	0	63.75
*EparIR6*	*Pachyrhinus yasumatsui*	ionotropic receptor 64a	WJJ63355.1	3 × 10^−33^	69.77
*EparIR7*	*Sitophilus oryzae*	ionotropic receptor 40a-like	XP_030764108.1	7 × 10^−35^	61.61
*EparIR8*	*Cylas formicarius*	ionotropic receptor 75a-like isoform X3	XP_060526926.1	5 × 10^−24^	62.16
*EparIR9*	*Sitophilus oryzae*	ionotropic receptor 40a-like	XP_030764108.1	2 × 10^−23^	63.75
*EparIR10*	*Pachyrhinus yasumatsui*	ionotropic receptor 8a	WJJ63357.1	9 × 10^−34^	48.53
*EparIR11*	*Pachyrhinus yasumatsui*	ionotropic receptor 93a	WJJ63358.1	1 × 10^−54^	68.18
*EparIR12*	*Pachyrhinus yasumatsui*	ionotropic receptor 21a	WJJ63354.1	5 × 10^−127^	78.76
*EparIR13*	*Pachyrhinus yasumatsui*	ionotropic receptor 93a	WJJ63358.1	5 × 10^−62^	60.71
*EparIR14*	*Euwallacea similis*	ionotropic receptor 25a	XP_066256016.1	0	86.87
*EparGluR1*	*Pachyrhinus yasumatsui*	glutamate receptor ionotropic 4	WJJ63362.1	0	63.72
*EparGluR2*	*Dendroctonus ponderosae*	glutamate receptor ionotropic, kainate 2	XP_048525692.1	0	88.28
*EparGluR3*	*Pachyrhinus yasumatsui*	glutamate receptor ionotropic 3	WJJ63361.1	0	67.75
*EparGluR4*	*Sitophilus oryzae*	glutamate receptor ionotropic, kainate 2-like	XP_030751235.1	0	62.98
*EparGluR5*	*Pachyrhinus yasumatsui*	glutamate receptor ionotropic 1	WJJ63359.1	0	73.84
*EparGluR6*	*Sitophilus oryzae*	glutamate receptor ionotropic, kainate 2	XP_030751944.1	0	91.87
*EparGluR7*	*Sitophilus oryzae*	glutamate receptor ionotropic, kainate 2-like	XP_030749785.1	0	69.54
*EparGluR8*	*Sitophilus oryzae*	glutamate receptor ionotropic, kainate 2-like isoform X1	XP_030751945.1	0	70.83
*EparGluR9*	*Sitophilus oryzae*	glutamate receptor ionotropic, kainate 2-like isoform X3	XP_030751453.1	0	84.78
*EparGluR10*	*Sitophilus oryzae*	glutamate receptor 1-like isoform X1	XP_030765898.1	0	84.6
*EparGluR11*	*Euwallacea fornicatus*	glutamate receptor ionotropic, NMDA 2B isoform X2	XP_066141506.1	0	85.9
*EparGluR12*	*Sitophilus oryzae*	glutamate receptor-interacting protein 2 isoform X2	XP_030749851.1	0	85.93
*EparGluR13*	*Sitophilus oryzae*	glutamate [NMDA] receptor subunit 1 isoform X1	XP_030759804.1	0	89.76
*EparGluR14*	*Leptinotarsa decemlineata*	glutamate receptor 1-like	XP_023011564.1	2 × 10^−16^	38.14
*EparGluR15*	*Zophobas morio*	glutamate receptor 3-like	XP_063905986.1	1 × 10^−07^	30.48

**Table 7:** Best BLASTX matches of *E. parallelus* SNMPs.

Gene Name	Best Blast Mach	Gene Description	Acc. Number	E-Value	Identity(%)
*EparSNMP1*	*Cyrtotrachelus buqueti*	sensory neuron membrane protein 1b	WAQ79968.1	0	56.67
*EparSNMP1a*	*Pachyrhinus yasumatsui*	sensory neuron membrane protein 1a	WJJ63366.1	0	61.48
*EparSNMP2a*	*Pachyrhinus yasumatsui*	sensory neuron membrane protein 2a	WJJ63368.1	0	51.42
*EparSNMP3*	*Dendroctonus ponderosae*	sensory neuron membrane protein 2	XP_019770844.2	2 × 10^−44^	56.03

## Data Availability

The raw data supporting the conclusions of this article will be made available by the authors on request.

## Supplementary Materials

The following supporting information can be downloaded at: https://www.mdpi.com/article/10.3390/insects16050504/s1, Table S1: Information of candidate chemosensory membrane proteins; Table S2: FPKM value of *E. parallelus* chemosensory membrane proteins. Text S1: The amino acid sequences of complete putative chemosensory genes in *E. parallelus*.
