# Supplementary material for: Transcriptome Analysis and Identification of Chemosensory Membrane Proteins in the Head of Euplatypus parallelus
Source: Insects. 2025 May 7;16(5):504. doi: 10.3390/insects16050504 (PMC12111860; doi:10.3390/insects16050504)
Supplement: Supplementary file 1 [file insects-16-00504-s001.zip › insects-3583138-supplementary.pdf]

Table S1 Information of candidate chemosensory membrane proteins

Gene name	Complete ORF	ORF Length(bp)	Number of amino acids	TMHs
EparORco	Yes	1452	483	7
EparOR1	Yes	1179	392	6
EparOR2	Yes	1194	397	7
EparOR3	Yes	1218	405	6
EparOR4	Yes	1230	409	6
EparOR5	Yes	1284	427	7
EparOR6	Yes	1290	429	6
EparOR7	Yes	1194	397	6
EparOR8	Yes	1191	396	6
EparOR9	Yes	1335	444	7
EparOR10	Yes	1173	390	6
EparOR11	Yes	1335	444	7
EparOR12	Yes	1143	380	6
EparOR13	Yes	1161	386	6
EparOR14	Yes	1182	393	6
EparOR15	Yes	1197	398	6
EparOR16	Yes	1167	388	6
EparOR17	Yes	1176	391	6
EparOR18	Yes	1137	378	6
EparGR1	Yes	1335	444	7
EparGR2	Yes	1203	400	7
EparGR3	Yes	1284	427	7
EparGR4	Yes	1335	444	7
EparIR1	Yes	1440	479	3

EparIR2	Yes	1650	549	3
EparIR3	Yes	1437	478	3
EparIR4	Yes	2822	925	3
EparIR5	Yes	1689	562	3
EparGluR1	Yes	2442	813	3
EparGluR2	Yes	2727	908	3
EparGluR3	Yes	2550	850	3
EparGluR4	Yes	2745	914	3
EparGluR5	Yes	2667	888	3
EparGluR6	Yes	2754	917	3
EparGluR7	Yes	2775	924	3
EparGluR8	Yes	2604	867	3
EparGluR9	Yes	2736	911	3
EparGluR10	Yes	3129	1042	3
EparGluR11	Yes	3177	1058	3
EparGluR12	Yes	2025	674	3
EparGluR13	Yes	2874	957	3
EparSNMP1	Yes	1617	538	2
EparSNMP1a	Yes	1551	516	2
EparSNMP2a	Yes	1527	508	2

Table S2 FPKM value of *E. parallelus* chemosensory membrane proteins

Gene name	FPKM-F1	FPKM-F2	FPKM-F3	FPKM-M1	FPKM-M2	FPKM-M3
EparORco	67.33	61.13	61.83	11.85	16.23	14.06
EparOR1	0.96	1.29	1.28	1.18	0.64	1.66
EparOR2	2.83	2.58	2.28	0.72	1.3	0.83

EparOR3	4.96	3.43	4.28	4.5	0.59	0.77
EparOR4	19.91	21.44	18.65	21.25	21.24	21.59
EparOR5	1.93	3.06	2.6	1.23	0.97	2.21
EparOR6	3.03	2.14	2.59	0	0.13	0
EparOR7	12.67	16.81	15.26	3.17	3.32	4.13
EparOR8	2.86	1.59	2.09	2.11	1.48	1.85
EparOR9	1.51	1.33	2.46	0.45	0.12	0.31
EparOR10	2.33	2.75	1.67	0.11	0.13	0
EparOR11	2.05	2.67	2	4.21	2.73	3.98
EparOR12	5.7	8.08	3.62	2.6	1.79	3.38
EparOR13	0	1.05	3.61	5.64	3.06	3.33
EparOR14	2.15	2.62	1.41	0.05	0.28	0
EparOR15	2.72	1.23	0.58	1.74	1.85	1.59
EparOR16	3.96	0	0	0	0	0
EparOR17	5.13	3.26	4.62	1.3	0.66	1.44
EparOR18	4.26	6.33	4.37	0	0	0
EparOR19	1.1	2.26	1.2	0	1.37	0.16
EparOR20	1.42	0.73	0.56	1.68	3.29	0.17
EparOR21	1.2	2.17	1.25	4.16	0.18	0.88
EparOR22	4.01	1.01	2.67	0	0.11	2.71
EparOR23	0	2.38	0	0	0	4.02
EparOR24	0.38	4.46	2.62	0	0	0
EparOR25	9.05	6.04	6.34	2.43	1.45	1.87
EparOR26	0.99	0	2.38	0.2	0.96	0.59
EparOR27	1.78	1.9	0.94	0.15	0.18	0
EparOR28	0	1.06	0.26	0	0	0

EparOR29	2.42	1.85	1.4	0.22	0.44	0
EparOR30	2.95	4.58	4.72	0.67	1.03	0.65
EparOR31	0	0.79	1.12	3.09	0	1.61
EparOR32	1.63	0.55	1.68	0	0.62	0.51
EparOR33	2.24	1.68	2.39	1.12	0.58	0.98
EparOR34	0.16	2.79	0.24	0.39	0.47	0.91
EparOR35	11	9.73	13.93	7.8	5.24	6.67
EparOR36	1.66	0.47	1.25	0.84	0.9	0
EparOR37	1.11	1.33	0.83	0.81	0.44	0.22
EparOR38	0	2.38	0	0	0	4.02
EparOR39	0	0	8.89	3.94	0	0
EparOR40	3.45	4.99	1.77	0	0	0
EparGR1	2.05	2.67	2	4.21	2.73	3.98
EparGR2	0	0.43	0	7.69	7.35	5.62
EparGR3	1.93	3.06	2.6	1.23	0.97	2.21
EparGR4	1.51	1.33	2.46	0.45	0.12	0.31
EparGR5	1.79	1.09	1.07	0	0.42	3.38
EparGR6	1.24	0.98	1.84	1.51	0.78	1.37
EparGR7	2.92	2.98	3.49	1.65	2.15	1.87
EparGR8	4.55	3.74	11.68	1.97	9.43	4.07
EparGR9	4.38	1.29	0.62	1.21	0	1.22
EparGR10	29.7	23.91	24.83	12.31	21.02	12.76
EparGR11	0.37	0.43	1.81	2	0.64	1.23
EparGR12	0	0	0	0	0	0
EparIR1	1.44	0.98	0.81	1.1	1.12	0.53
EparIR2	2.42	2.43	6.12	1.79	2.2	2.17

EparIR3	0.53	0.87	0.58	0.46	0.21	0.46
EparIR4	6.96	6.96	6.83	1.07	0.97	1.37
EparIR5	16.04	15.46	14.14	26.64	28.66	25.45
EparIR6	5.01	4.63	4.89	7.01	7.91	7.79
EparIR7	0	0	0	0	0	0
EparIR8	0	1.41	2.06	0	0	3.17
EparIR9	0	0	0	0	0	0
EparIR10	0	0	0	0	0	0
EparIR11	7.01	0	3.02	0	0	0
EparIR12	0	1.81	0	0	0	0
EparIR13	0.6	1.43	1.2	1.29	0.71	1.21
EparIR14	0	0	0	0	0	0
EparGluR1	30.91	22.17	28.65	18.86	14.97	18.46
EparGluR2	4.01	2.97	3.08	2.25	2.68	2.51
EparGluR3	1.48	1.66	1.97	2.2	2.16	2.29
EparGluR4	25.7	24.06	23.9	38.92	36.02	38.69
EparGluR5	15.51	12.42	14.31	32.24	31.76	32.08
EparGluR6	12.01	12.27	10.94	11.17	7.69	10.99
EparGluR7	17.12	17.01	18.37	35.22	31.69	34.86
EparGluR8	361.27	364.17	403.49	645.89	572.37	658.02
EparGluR9	7.53	9.9	7.49	3.93	3.43	6.16
EparGluR10	17.28	22.34	15.57	22.54	15.3	19.63
EparGluR11	1.34	3.27	3.76	4.6	3.76	1.89
EparGluR12	9.2	9.39	9.04	8.09	8.59	9.28
EparGluR13	1.32	1.78	1.25	2.1	3.11	2.46
EparGluR14	0.45	0.24	0.38	0.21	0.55	0.43

EparGluR15	2.32	1.6	1.27	1.96	2.16	0.9
EparSNMP1	16.36	15.92	12.45	8.15	6.51	6.06
EparSNMP1a	6.14	7.33	7.89	2.07	1.65	3.44
EparSNMP2a	1.85	2.93	1.15	0.72	0.49	1.01
EparSNMP3	2.28	6.52	2.97	6.37	5.19	10.05

Text S1 The amino acid sequences of complete putative chemosensory genes in *E. parallelus*

OR

>ORco

MMNKFKVAGLVADLMPNIRLIQASGHFMFNYHADNSGALHTLRLGYSCCHLFFVLMQYGCIFANLIRERDHVSHLAANTVTVLFFTHCITKFLYFAARSKLFYRTLGIWNQANSHPIFVESNNRYHAISLKKMRQLLYIIVSGTIFSASAWTVITFFGESVHYIKDPDNMNETITEPIPRLLVKSWYPFNAMSGPMYFIALIFQIYYVFFSLFQANLLDSLFCSWLIFACEQLQHLKEIMKPLMELSATLDTYVPKSADLFKAPPSAGSQDNLVDNDFNSKKEDYDLKGVYSTMKELGNLNFRSGALQTFGQGGGGLVGPNGLTKKQELMVRSAIKYWVERHKHVVRLVTAIGDAYGVALLLHMLTSTVMLTLLAYEATKIDGVNVYALTTVGYLLYALAQVFHFCIFGNRLIEESSSIMEAAYSCHWYDGSEEAKTFVQIVCQQCQKALSISGAKFFTISLDLFASVLGAVVTYFMVLVQLK

>OR1

MGDKKTKQFFNTGKFLLKLCGLWSYDIKNRYVKILYIIYRCAMEFSFAFSICSFFMSAIMQVKTNVTVAIECSSRVLFMIMVAFKMKFVQRKEMRHMVELLVQEETKTISIDNEYIRKVYNKHVIVARSMCTTILLFLYGAGYWTLSDSFKIRKLNDNLPPENQITDGLPFWYPFDKESHVAFVVMAEFGHVGFALAVNSSIQVLINSIMIFLRAQLKILQYNCRHFDEIIIVDGRIEESKDVLKNLRQLVIKHQQIIEIVRDFNSSFKNILLAEYMLTSLQFASTLLSLIQGIKVQFNSTFFMHCLLMLLALAWNANEIELESSSSLSRALFESNWYLYDKKCNSFIRLMMVRCIRPLFMSIGPLGTMNLDAALSRLKLTYSVLSVLQGRT

>OR2

MTEKIQILRLHIQCLKYLLLWPKDNLSKKQNTILNYGYFCLSTSFSIPVVCAAGYQLYVGVEDVNILVEALIAVCDIIGYLVVYYCFLRDYELVENLLDGINVFLEFCDRKLILDIDAKCITYTRYLLWYVTIGVTINIIWPLISVQSCINSRISEFYVKHDPCGMPTQNFYFFDASKPQYFWIVYLIEANYCYHICYAFTLATAIATGFLMHIIAQLRNCGKIFEEIFDSEIHSNEKEFEKRFFKCIKYHQTILNYAEKFFYDFSNMLIIYVTMTSFTLAVIGYQIANSETKLHDRLRYTMLLVGWLLLFYLVCHYGQKVKDESQQIAESIYNSKWHKSTANLRFRKYLVLIIARSQKPLTLKAKYMGTISLDRYVAVLKTAYSFFTLLISVTDDN

>OR3

MEFPHNKLLKISMRLIAGIGGWKLFKWKNNKIQILYKCYALISHYYFLEYIARSYFKLILLLMAQHLDVEEILGNMCITLLYTISFVQIRTFKSDKLKDLFNQM

IETEEKILNDGHPEIQKIYMGYVKYNIRCNILFLINGWMVSILYYVRPFCIEPEIILGNNNETLIKRPMPLSTWWPIDPYKNYMVAYWYNICEAILGSSYVISSN
LMTFSLIIFALSQLEIVNWKAKRFPLVKSENDKRKVVTTRDAIKAEFINLIEDHKKVVKYVDTFNAAMKYVVLFDFLQCSLQLATITLQLLVMEINIQNCIFVG
EFAITMLIRLTIYYFNANEIIVKSENIAHSIWHSDWCNLDDDMKKMIVIFMARTQRPLNLLIGPFGIMSLDTFIRILKATYSYVMIFTNTNN
>OR4
MSRIYPKTDHLRFPMKAASLIGMFPTDLMFRGQKTFQRIYHYYYSAILFYFTTFLVTAYIKLIILLQDEKINMTDVAENLCITLLYTNSLIRQMIMKFNKGFRS
MLQQILDTEQGIQDSKDKKKIELLNKCSESSNNHCRLYLIIVFTTAALYIAKPLFHSGYPMVIKNVTYQIKPLPLSSWIPFDRRTHFKMAYLCQIVDSVIGAQVL
VYTDLVMFSLILFPTGQLKIMGHLLRNYKQYKQQIKLKFNIENDEHAAFVTFKGFVLEHRKVINYINQFNNTMSTLTTFDFLQSSLQIASIVIQTMGNKITLW
YVLYSSSFFVGMFYRLILYYYFPNDIILLSDDLTTAIWESDWYNEKQSIKTMMHIVMLRTQLSLKLFIGPFGVMSIDAFISILKATYSYIMLMYGVH
>OR5
MNPLREMELKDLADLYGEELHIKSLPKVWGSPRSTKIAKRLTLDQNNGEIIDEHDQFYRDHKLLLILFKVMGVMPVERGEIGKITFGYTSKPMIYAYIFFAVT
SVLVVLVGYERFDILLNKSKRFDEYIYAIIFIVYLIPHFLIPFVGWSVAFQVCDYKNSWGTFQLNYYKITGKDLEFPYLSTLIGVISLGCLFLAVGFLLTLSVLLE
GFTLYHTTAYLHIITMINMNCALWYINCRAIGNASSAVAENFRKDMTEYCSAYIIKYYRILWLELSEILQKLGNAYARTYSTYSLFMMANITISVYGFTSEVVD
HGIKFTFKEMGLIVDGVYCILLLFIFCDCSHNASSNIAERVQWTLLEVDLENVDQNAAKEIQLFLIAIQMNPPKVSLKGYTVVNRELLTSSIATIAIYLIVLLQF
KISLVNLKG
>OR6
MKRKTIGKNSIDNYPENCFIATDIFLIICGQGKFGKNTSWVLKRLYSFYNVLLIFITIQFVIFEFVEFRNSFTDIPLTIRRIAMMTTHLLGLLKLWVLISKGDKMEI
IKEKLQNKQFHYDSVTDCFQPSQSMHKARKFLIRLTVFLGCLYSFVGVSAHISSTLTVSKFTIGQEFVNGTSCDLIVPYNYYSPWKRITKTTCNISLVYMDISL
NIFAWYIACFDAILVSFLHILKKQLDIVQEALITLRQRCCIKLSLNKNFKVFSDLDYPPIEKKMYKEMLHCIRHLNLLLDVRNDIEDAFSQMALVQTLSSLFIFA
SCLFTVAREPKSSPNFFSQLQYFFAVLSQFSVYCWFGNEITEAGQSVPLALYKTEWIGSSSRFKNLLLFTTVRMQRPIFVSIGKFTPLTLSTLISVIKGSFSYFTLF
QTFGED
>OR7
MADNVKLIRTAKKLLIFGGLWSIPFSTNPKLNKLYGFYMKGFLTCLIVVQIAVIMELVRLVTVGGEWEKTLVAFVVFVNGLKILIRVIIYKVYKVPDLLQNIID
KEKLILKSKDAEIRKTYFNYIIYCERMTKSLLVLTSFVICIYLYIRSRECIEANEIHKALNESLVPPDMYPIYFPNAAEHLTIVFAMNYCVGTFTALYNPGSIMVL
LGCLIYIASQLEIHNIKLSKVHIIAKDEYNGNAAAALKTLIKEYEEIKKFVETINDSCKYIILMEFCLSSLDCAGVAAQLVDSREKKLIEYIFLTSYTILLVYQIFII
SWTANEVQYHSSEISVALSKCNWYEFDKEAKQILCIMMTRAQRPLFITIGPFGPMTTQTFVLEEAAPNLLENIFLNIRV
>OR8

MSQHSSNVLDFFKIHQIVFFYTGFWKVKKFTKLYRCLTVFVMITNIFLIFPPLLYLITNEVDLFEIVNSWYIICTFGITIVFSLEFIYRFDDVLEVFEMTKREPLFQ
IQSTQHYKIAKKGRFISKCLHYYAIFITSLTNIFWILRPSFEEGKNLLIKGVFPYNTEISPYYEITRILQSFSSIIVVIHTVNIDIYAANSLIYISVQCELLKYTLEHLN
EFQTIHGQLRQTGRVLHGNDDDYCCEMLKNIKMCFEHYVQIKRMAKLVERIYSVSLLSLLIGSLLTVCTMLYQIVLVPIGSNDFFYLVFFLICLLSQQAIICIIG
AEVTARSNKLTLSIERIPNWIDCDQNFHQIMIVFLLNLEKPIVIMAGNMLPLSLQFLKTLLLNSYTYFAILTDM
>OR9
MFNNEKQRRASETIRISTSDSNNFNYDGNNNNNNNKEKNGGKLKEPDPGLLDKYDNFYDTTKSLLVLFQIMGVMPIERQKGKTVYRWFSPVTCWAYFIYM
IETLYVTIVFKDRLTLILEAGKRFDEYIYGVIFLSILMPHFLLPVGAWTNGSEVAKFKNMWTKFQLKYYKVTGRPIIFNRLTEITYALCITSWLAAISIMIVQHCL
QPDMLLWHTFAYYHILAMLNGLCSLWFINCTAKGRVAGWLGDYLQLALQSKNSANKLADCRELWVDLSHMMQQLGKAYSGMYALFCLLVLLTTIVATYG
CLTEILDHGISFKEGGLFLISFYCITLLFIICNEAHSVTRRMGPEFRERLLSINLLAVDRDTISEINMFLNAIESNAPIMNLNGYANMNRKLITTTITSSATYLVML
MQFRLTLTRNAAFEARKANIMAVVQEAVHPDK
>OR10
MEIGDEMFNICKKMMKVCGIKYDESQRPKIFYEIFQKVFFITSIITFIVFNLNFPMHLRKHGLNDDVFFPLAATVMSINELVNQINFRSENLRKLINGAISETNK
CSRSDPKIKEIYKKYHRYASILNKLLIIFQPIIAASFYTQTFRKGLTCLSPTATQNDLKAFLKYVVIWIPFDIEKNILATLALSGIVYFCTAILILTVPIMFNTVILNA
VAQLEVLQYQFETYDLDLPETDSREDIEDALERKFKQLIKRHQNIIEHIQYLNESIKTSILVNVVFPIVIFAVMLARLLKGMYILGGLLLISAAKLIVIGYLANEI
NTQSLELAGKLFKSEWHKRSQSIKKMILIVMMRSQRPLTLDIGWFGPMTINTSLSVLKTAYSYFTVMYQR
>OR11
MAPVSITIADPQTYRNNNDPYNSSTINFPRRKSEAIKIESSKSNKRDLDFNLLETHDEFYKATKSLLVLFQVMGVMPIQRNKGHTTFKWTAPVVIWAYFLYTG
ETILVSLVFNSRLNLVMQPGRAFDEYIYGIIFLSILIPHFLLPLVAWSNGAEVVKFKNMWTRFQLKFVQVTGKPISFDKLKSTCHFLYIFSWTAAILIMLSQYCL
QADMLLWHTFGYYHILAMLNGLVSLWYVNTRAMGKVATWLSELLTEALHLDENSAEKLADYRDIWVDLSHMLQQFGKAYSGLYACMYLLVPLTSIVATF
GCVTEILDHGISLKEGGLCIIAIYCCIILFIFCNQAFHASYKMGSEFREILMNVNLTLVDDRTVQEINMFLTAIQKNPPIMNLNGYTEIDRKLLSTTATSVVTYLVI
LMQFRMTIMKNNARSTRHNATLSLYNTTFM
>OR12
MVHEIPSFLNGEQIRLGILGFYPKRIKRNFFLILIMILVYLVTFIQMLTCAAFVIIVSELTQCTEAFVFLMTQIGFINKLINLHSKLNNIFTLENMMADKILLKVDK
KEQSILDGYFQSCQKVLNIFNVLCFLVLVLFAAFPFIDINESDTKYYPLKGKFPFNPDNYYLFVYSIQVLSVAFSAWINGSMDCLFTKHVVIATAYFEILYEKIL
NVSNNVTDYNDGEIERRIKHCAIYYNKIAKIAKLIEKTFSHGILVQFICSGIVLCLTGFQLILVVPFPSGRFGLFTTYLLCMMCQPALYCWHGQILMDKSNEITR
ACYTVDWLNMNVKCRKMLITIMERAKRPVVIKAGGIFMLNIEAMMTILRTSYSYFVVLQQIYE

>OR13
MVTSLLVPKFFLVISGMCPVDVIPAKIKILYIFYACIIQFSFIIISVLSPMEIIRLFYTHAEAVRKATGCATYLTILLIVAKLIIYQKYNVPKTNFTCSKYEEDIKKQN
DEDIKKVYNEYFKFNWVVDMWTVIITFLVVATFIGLGLQFIIQTGLKNIDLYRIAVFYEIYFWFDRNKYAEFIISYNVFLMILGFVFNVTCQTTFYSAINYGTLR
LKVLRIQLGKFETYTEKDPVDTLKDFIKEHQDIIDYIHYLNDSTKHVILFEFLLNSFNVALVMFHLATGTELQIIIFDILYFSMLISQIFLLGWVANEVKVQNFAI
ADGIAESCWYEQSPQVKQMMHIMLMRAQNPLTIDNGVFQPMTNETAIMIVKASYSYTTLMLNDYQK
>OR14
MEIGDEMFNICKILMSLSGIKYNENHNPNLIYKLNQIFFYIASIIAFFSFHFHFPMYMTMTKNEYDEDVFFPLAATMVSISELINQINFRSKGVRKFINRAISETN
KSSRTDPNIRKIYEKYHRYCNILNRSLIISLLLIEICLYTQVVRTGLKCKNPTATTKDLKVFFSYIVVWIPFNIKDNINATLVAIAFIYYCFAMSIITVVIIFNTVILN
AAAQLEVLQYQFETYDLNLPKTDKRENIEDVLEKKFKKLIERHQNLIIHIQDLNESIKISILINVIFPIVIFASLLGQLLKGQYIIGAFLLFSAAKLLVTGYLANEI
NVQSLELACKLFNNKWYERRESIKKLILIVMMRCQRPLSVDMGWFGPMTINVSISVLKTAYSYCTVMYEKN
>OR15
MQEEVGDKMFKFTIQLMGIAGFRKSYNSHARWGFIYKYYSFIMRKIYIIGILLFLIQLIRKTFTHEIDDTLMILLMGVSGFTVYYMKTAPSCSSEMWTLMSES
KTKANQLAKKNSIYGKIFLQYHKYNYSFILSAVISSVGAVILHTISVHLQQRELEKKMLSNSSHYEGSAILKTVWLPLNTKECGNLYVILSSLIVASMDILVSA
NYAVFNTILIYSIARLKVLQHQFKTYDTFDTDGMDAENIEKIAVDKLKFVIKEHQEVINQIRRINQQVKSPIFIQTTLSSTMAAVNLSQIVLGYNILFNMCFILAT
LFPFAVTSYMANEIKFQSVELGTSMYKSNWYMRTPLTRKMILIVMIRCGAPLRIDNGQFGPLTNDTAVRVFKGVYSLTSFLTSTT
>OR16
MIEEENAPIRRNFHNFLKFDFKMMKVFGIWLDIENPPPKWFRVYACFFHLLLFYISSFLTSVKWYLKSTDIKSFAMVGQLVMLIFLVHIKSTILVATASKVSKLA
KILDVEEFQSRSDEEEKITLKVWKMCRKVKLSIFIGIYMSVIMLLILMSVIKRADYIPIPVWYPFDMKSLGHFELVYLHQFIMALTFGTFNILHDTIMIANFIFIC
LQYDILCIRIKNMKENPTEPNDVSFKDCVKHHLKILEFHKIFECTLGKTLFVHLACISFSMCMILFLLTNLEKNSVDFYFALTLFVSVILLLFIPCWFGTEITIQSE
KPPVALYDFNWLDCSISFKKDVAFFLLHLQRPIKVYALNYFEISMITFAKIMKSSYSYLALLNNLSAKQK
>OR17
MMNNNTKLIKMAKFFMILSGIWHHRLSKNYRIINVLYYIYSFIIIISLVLFAIFMIVEQIRLVSQNYPLELIWASIGIVINAYKIIVKLIVLYKWKMHKLFDEVVSN
EENIWQSEDEDIKTLYRKKVKFCKYAMIIIAVWLLLTIILFVGTGITGDNKLIKYNQSHNDTLESHSMFQFYFPVNKLEHQTLVLSINMIFSWMAWVTNSVSHI
VFLTLIIYAAVILEILQIRIRKEIKPYETNKETVQVIRELILEHIRIIDYVNHLNNCIKYIILLEFLLNSFDTATDIISIVNAKSVEALWLVWYVIQQLFQIFLLAWNC
NEIQIQSLQISDSLFESDWYLLNNNEKKLIQLMMVRAQRPLSIDIGPFGPMTTQTALLIIKGAFSYISIMK
>OR18

MSDRKNYKNFIKFDCTILKMIGIWLDIENRPPIWFRVYALIAHALLIGTISLTLVNWCLNSTNIVNFAIFGQMVIMLSLIQFKLITFVINLRNLLKLMEFFDEPEF
QTKNKTEEEILFQTWKMSKKVKLSIFFIMYMSLIMAVTLMTFTERGEYLPIPMLYPYDIKPFGYFELVYLHQTVFGCYAATFYSLSDTIMAASFIFIGLQSDILC
IRIKNMKENPIEPNDVSFKDCVKHHLKIKEFHQLFSTTFSKILFYLLACISFSMCMNLFLLTNLKSDSMDFHLSAAMLMSVTGSLFIPCWFSTQIEIKSEQLPVV
LYEFNWLNCSIQFKNDVVFFLLHLQRPLKVYALGFFEISIVTFTKIMKTSYSYLAVLNNLNE
GR
>GR1
MAPVSITIADPQTYRNNNDPYNSSTINFPRRKSEAIKIESSKSNKRDLDFNLLETHDEFYKATKSLLVLFQVMGVMPIQRNKGHTTFKWTAPVVIWAYFLYTG
ETILVSLVFNSRLNLVMQPGRAFDEYIYGIIFLSILIPHFLLPLVAWSNGAEVVKFKNMWTRFQLKFVQVTGKPISFDKLKSTCHFLYIFSWTAAILIMLSQYCL
QADMLLWHTFGYYHILAMLNGLVSLWYVNTRAMGKVATWLSELLTEALHLDENSAEKLADYRDIWVDLSHMLQQFGKAYSGLYACMYLLVPLTSIVATF
GCVTEILDHGISLKEGGLCIIAIYCCIILFIFCNQAFHASYKMGSEFREILMNVNLTLVDDRTVQEINMFLTAIQKNPPIMNLNGYTEIDRKLLSTTATSVVTYLVI
LMQFRMTIMKNNARSTRHNATLSLYNTTFM
>GR2
MALILNGSNSKNRLKVKSSLTIIAQIIINLLNYKWFTFFYSIANIAFQCYAFITTIFYMSFLKNKFHLLPYLNSQVAVILLTLTNFITIRCMWKCPKFYQNLNNTIR
PVGSDRSNKTDNTFRNWLIMYHSYFIICTSINCYLILTVLNVKAYYKLHFVRHVQWFCLISSLLVVLEFADNIRRMFVEINMALEKIPEKYLLQKYSKLGTKS
LSGIFTIHPDENACECLQDIKYLCKYHNDICNILDEFNNNFGLLFLAFTINFIAGTLWTVAFLLSLGFSSYRINVVTLSKWVFFCEVTCLLYTLSGTILSAFMGS
KLAKEANKTSTICYVIVNTLKMEENRNSNLLRDQLLLLAKQVKIRNPVLDAAGFFFVDVNIFNLVTSNIVTYCIVELQFLINNQY
>GR3
MNPLREMELKDLADLYGEELHIKSLPKVWGSPRSTKIAKRLTLDQNNGEIIDEHDQFYRDHKLLLILFKVMGVMPVERGEIGKITFGYTSKPMIYAYIFFAVT
SVLVVLVGYERFDILLNKSKRFDEYIYAIIFIVYLIPHFLIPFVGWSVAFQVCDYKNSWGTFQLNYYKITGKDLEFPYLSTLIGVISLGCLFLAVGFLLTLSVLLE
GFTLYHTTAYLHIITMINMNCALWYINCRAIGNASSAVAENFRKDMTEYCSAYIIKYYRILWLELSEILQKLGNAYARTYSTYSLFMMANITISVYGFTSEVVD
HGIKFTFKEMGLIVDGVYCILLLFIFCDCSHNASSNIAERVQWTLLEVDLENVDQNAAKEIQLFLIAIQMNPPKVSLKGYTVVNRELLTSSIATIAIYLIVLLQF
KISLVNLKG
>GR4
MFNNEKQRRASETIRISTSDSNNFNYDGNNNNNNNKEKNGGKLKEPDPGLLDKYDNFYDTTKSLLVLFQIMGVMPIERQKGKTVYRWFSPVTCWAYFIYM
IETLYVTIVFKDRLTLILEAGKRFDEYIYGVIFLSILMPHFLLPVGAWTNGSEVAKFKNMWTKFQLKYYKVTGRPIIFNRLTEITYALCITSWLAAISIMIVQHCL
QPDMLLWHTFAYYHILAMLNGLCSLWFINCTAKGRVAGWLGDYLQLALQSKNSANKLADCRELWVDLSHMMQQLGKAYSGMYALFCLLVLLTTIVATYG

CLTEILDHGISFKEGGLFLISFYCITLLFIICNEAHSVTRRMGPEFRERLLSINLLAVDRDTISEINMFLNAIESNAPIMNLNGYANMNRKLITTTITSSATYLVML
MQFRLTLTRNAAFEARKANIMAVVQEAVHPDK
IR
>IR1
MGFDYEFVEPTDGTFGEKINGTWNGVIGGLAYGDTDMALTALIMTADREEVVDFVAPYYENTGISIVMRNPVRKTSLFKFMTVLKLEVWLSIVASIVITGVLI
WFLDKYSPYSARNNKEAYPYPCREFTIKESFWFALTSFTPQGGGEAPKALSGRTLVVAYWLFVVLMLATFTANLAAFLTVERMQTPVQSLEQLARQSRISYT
VVKDSEIHQHFINMKFAEDTLYRMWVELTLNASTDDTRYRVWDYPIREQWGHILLAINDSNPVANASEGYRLVDEHLDADFAFIHDSSEIKYAISKNCNFTEI
GEVFAEKPIAIAVQQGSHLQDDLSKIILDLQKERFFEELQAKYWNHSAKGYCPNSDENEGITLDSLGGVFIATLAGLLLAMITLVFEVIYYRNKAKKLKEEKK
QAAKTGASKIQSFKPHKVHNIASNTDLRRKIATISKPYKPPIMVMERNENVSEHSLYPKARNRIHRIN
>IR2
MFVLDISCDFSNDLLKKANEARLISAPFHWLIISRKFLHSYEIQEMFSKLLILVDSDFNLCEIYSNETVVFRKIFRKHKTDSKISVERFGTWSLPKKFKKDINFE
LMLAERRKNLGINLNACLVLTNNDTIKHLTDRRDKHIDSIAKVNYALVLRLASIYNISFNFSVTNTWGYKNNESKWTGMMGELTEKRADVGGTSLFFIKERI
DLIDYIAMISPTRSKFVFRQPKLSYIMNVFTLPFDTKVWFSSFGLVLIMILALFMVMRWEWYKPHVIEINDKSSITELDYSFIETSFTIFGALCQQGAATLPHSIP
GRITTIFLFISLMFIYVSYSANIVALLQTSSNSIKTLEDLLDSRIKLGVDDTVYGRFYFMTAVEPIRKAIYEKKVAPPGKPPNFMNLTEGVHRIKEGLFAFHMETG
VGYKLIGEIFREDEKCGLQEIPYIQVVDPWLAIQKDSPYKKMLKIGFRMIQESGIQQRENIIIYTRKPVCTTKLSTFFSVDLVDCYFTVAILGSGLLLSGMMFIL
EHLYKAINTRHSCDQKGSCKTEILN
>IR3
MTDQLFPHIAHGFRRKTLPLVSFHNPPWQILKLNESGDVLEYKGLVFDIIKELAKNLNFTYTIEVIKTNNWKSNITKFQSSYTNDSIEFMEQIDNLDVSTYKIP
QQILEMVHNKSVAMGACAFTITEANKQLINFTDPISIQFYTFVVARPKQLGRALLFISPFASSTWLCLAATVILIGPILYLVHKLSPVYEYNGIRSKGGLATIQNC
IWYIYGALLQQGGMHLPQADSARIIVGAWWLVVLVIGTSYCGNLVAFLTFPKNDIPITTLDELINHKDTVTWSFAKNTFFEAQLKATNDITQKTIYKESKDIKN
KRQMISKIKEGKHVYIDWKIKLQFIMKQQFDENGACTLDLGTDEFLDEQIGMIIAPDSPYLKKINAEIKKLHQVGLIQKWLEDYLPKRDRCSKRKRFVEVNN
HTVNLDDMQGIFFLLAFGLLISIVLLLIEKFRYKKNPKSQCQEKPITTIYNLIITQIAYRVL
>IR4
MRLNAFIFLFFCSVLFKQSLAQTTQNINVLFVNEEGNEIADKAIDVVLNYIKKVSKLGISVELKRVVGNKTDSQTVLDSLCKVYQSMLDSNNPPHLVLDTTR
SGLACEIVKSFTSALGLPTISASYGQEGDLRQWRTLKDNELEYLIQISPPGDIIPEMVRTLVLNQNITNAAIMFDNTFVMDHKFKSLLQNVATRHLIDEISYETA
KLPEQLDDLVRLDLSNFFILGNLENAKNVLEAAETKNLFNRKFAWHVLTKDKGDLRCNIKNATVLFAKPVVNPLFQDRLGTIKTSFQLIAEPEIDAAFYFDLA

LKSFLTIKEMLMDGSWKKNNVTNYITCDDYEAKNSPKRYNLDIRSYLQKDLPEPPTYGPFSISSNGLSYMEFSMALSAVFIRSGSSDKSNPLGVWQGGFDNN
LTLYNPREMKNFTADVVYKVVTVVQKPFIIKDESAPKGFKGYCIDLIDEIANLLHFDYEIEAVGDGKFGNMDENGNWNGVIKDLMDKKADIGLGSLSVMAE
RENVIDFTVPYYDLVGITILMKLPETPASLFKFLTVLENEVWLCILAAYFFTSFLMWVFDRWSPYSYQNNREKYKDDEEKREFNLKECLWFCMTSLTPQGGG
EAPKNLSGRLVAATWWLFGFIIIASYTANLAAFLTVSRLDTPIESLDDLSKQYKIQYAPINGSSTMTYFQRMADIEARFYEIWKDMSLNDSLSDVERSKLAVW
DYPVSDKYTKMWQAMKEAGLPATLEEAVERVRQSKSSSEGFAFLGDATDIKYLEMTDCDFTTVGEEFSRKPYAIAVQQGSPLKDQFNTAILQLLNRRDLERL
KERWWNKNPDRKQCEKTDDQSDGISIQNIGGVFIVIFVGIGLACVTLAFEYWWYKYRKNTKITTVAEAPQQPKSLSFPRDFGKMQSRRDEDFSGTIKKLYPK
PKF

>IR5

MGLAEIVLAGLCLNSTLNCTNDTDYHYDTQLAKYKRGLLESLKNEHFVITTLKNGPLSGYENTNGTLIGTGVAFDIIKILQREYQFKYKLVTPDDDTFEAPD
GTTGGVKNMLLKRRVDIAAAFLPMKFTNEFKYSKSLASAQWVVLMRRPKESATGSGLMAPFTATVWTLIIISLFTVGPILYLVMLLRGKICEETGEMVFSLP
ACMWFVYGALLKQGSTLNPTTDSSRILFSTWWIFITILTAFYTANLTAFLTLSKFTLPINVPEDIGKKQYKWLTNKGNGIIEELRNSREYKSNDGKSLYEQIGLP
KRQVNDDDNLILSDFVSKQNFMYIREKIVLETTMYEDYKQKTRANIEEAKRCTFVITNFPICTFKTAFAYRPGFKYAKLFDSTLQHIVESGITEFKQREFLPDT
KICPLDLGNKERRLRNTDLSMTYMIVGGGLIISTIVFIVELFIYYYYLKIRKGYFQKKRSLNLRQNGLTGKVTFNKKTTDYNLFEPIKHFITPPPPYHTLFGPPP
GVPGIDYTKKMINGRNYWVINDKNGMKTLIPQRTPSALLFQFTN

iGluR

>GluR1

MKQFKVEILICIFFVWIYLCSAKDSIRVGIFFTETDKDTEINWDIINNNLEQENAIDLPTPEITIVPNNDIFESKNILCNFIESLNQTRLIALFGPEEQLLSDALESV
SSHLNVPYFLTSWKANNNVHFENVFNMYPDSHLLAEGLAKIVNSFVWDEWGILYEDNDGLFRLQEVLKLQKYKTGAHKNLIYLQQLNDVDNRSILKKLR
HNSISKIILDCHVNRIMDILNQAQEVGLLSDFSTSFFLTSLDAHTLDYSGLKTLSNITTVRLFDPTQVKFREIVEEYFPNVPPNQIKVKTALIHDAQLLISQTINS
VLNKKTDLIPNSLHCTSLEKHLDELNISTTMQTLELNEGTLTGSISLLNGQRTTFNLDVIEIDKPENPIAKWNSDMPDKIYLTRNATEREYELLKRMQNYNFIIT
SRIGPPYLYERTDSEVSGNRKYYGYSMDLISEIAKIMNITFEFKITAANSYKNLVNDLIDRRADMGICDFTITPQRREVIDFSMPFMNLGIGILHKNLDSEEVNN
MYAFMRPISWTVWFYITTLNFSISMTMVVVARLATKEWENPKPWDSESKELENIWTLNNFFWLTMGSITTQGCDILPKSSPTRIITGFWWIFSLIITSSYTANL
AAFLTMGKKDVTVDNVEELAAQSKIKYGLLAQGSTEAFFSNSNNTLYQKMWNTMKNEKPTVFESDNAKGVERVLSTKNALYAFFMESTGIEFIMERNCDL
RKIGNLLDSKSYGIGMPMNADYRHSVNKAILRLQESGHLLRLKEKWWKPLEGEASCKWVHSIQFIQITLKLFFFCILRSLRISLIL

>GluR2

MASEDNGLIALFGPENQDLSSTLESACNHLNIPFILTGWIQRNSKEIETTFNLFPEASLLAKAYARIIESLDWDSFFVLYEDEEGLIKLQDILKLQIYKEGETKNS

LMLRKLEVSGDNRHIFKSIKETTISRIVLDCKTENIIEYLRQAKEVKLLTDFTTSFFLTSMDAHTVDFSPLNTKSNITTVRLFDPSTPNFQQIVNEFYPTLDPETIT
VKTAMMYDGMNLLSEAINAIKSENPDIIFEPLSCNETEQLTDHYGLIAKMHEIDIPDALTGKIHFENGKRDSFELQIVEFDKLDQPIGIWSSDEPDSIHLTRNAT
EREAEIQRRLQNHNFIVTSRIIAPYLFKNEDPEAKGNAQFYGHSMDLISEVAKILNITFEFRLTKDSSPKQLVIDLMERNADLAICDFTITPERREKIDFSMPFMT
LGIGILHKKAEADEEVSMYGFLGPLSTTVWIYIGALYLVISIILVLIARMSTEDWENPHPCDPKPSELENIWDFKNCCWLTLGSITTQGCDILPKGICSRIATASW
WFFSLIITSSYTANLAAFLTMSKIDDTIKSVEELATQSKVKYGCLDGGSTSTFLAQSNNSLYQRMWNIMQNEKPSVFESSNAKGVDRIQSTKNGLYAFFMEST
GIEYELERKCDLRRIGNLLDSKSYGIGMPMNADYRHSINAAVLKLQETGKLIELKAKWWKEHKNPCPTEVTENSDALALENVGGVFVVLSVCIGLAFLIAIL
EFLWNVHNVAVEEHISFLAALKIEVKFACNIFITKKRVKPLLSEAASSKSDVDMDGGDDNGNVGIIDTAASLLSVHDGGGSNRRTVSQSRTNLNVEEFSNGM
RKSKSKSKSKTNT
>GluR3
MASEDNGLIALFGPENQDLSSTLESACNHLNIPFILTGWIQRNSKEIETTFNLFPEASLLAKAYARIIESLDWDSFFVLYEDEEGLIKLQDILKLQIYKEGETKNS
LMLRKLEVSGDNRHIFKSIKETTISRIVLDCKTENIIEYLRQAKEVKLLTDFTTSFFLTSMDAHTVDFSPLNTKSNITTVRLFDPSTPNFQQIVNEFYPTLDPETIT
VKTAMMYDGMNLLSEAINAIKSENPDIIFEPLSCNETEQLTDHYGLIAKMHEIDIPDALTGKIHFENGKRDSFELQIVEFDKLDQPIGIWSSDEPDSIHLTRNAT
EREAEIQRRLQNHNFIVTSRIIAPYLFKNEDPEAKGNAQFYGHSMDLISEVAKILNITFEFRLTKDSSPKQLVIDLMERNADLAICDFTITPERREKIDFSMPFMT
LGIGILHKKAEADEEVSMYGFLGPLSTTVWIYIGALYLVISIILVLIARMSTEDWENPHPCDPKPSELENIWDFKNCCWLTLGSITTQGCDILPKGICSRIATASW
WFFSLIITSSYTANLAAFLTMSKIDDTIKSVEELATQSKVKYGCLDGGSTSTFLAQSNNSLYQRMWNIMQNEKPSVFESSNAKGVDRIQSTKNGLYAFFMEST
GIEYELERKCDLRRIGNLLDSKSYGIGMPMNADYRHSINAAVLKLQETGKLIELKAKWWKEHKNPCPTEVTENSDALALENVGGVFVVLSVCIGLAFLIAIL
EFLWNVHNVAVEEHISFLAALKIEVKFACNIFITKKRVKPLLSEAASSKSDVDMDGGDDNGNVGIIDTAASLLSVHDGGGSNRRTVSQSRTNLNVEEFSNGM
RKSKSKSKSKTNT
>GluR4
MAMFTNKIFIILILHYYSSLADNDIKIGAFLNYDLYAEASEATNWKAIQENLPGSFTYHTPDVEIVPDDESFETSKLFCQYASISPGYLFVFGPRRRINSETIESIC
SNFRIPYFITNWIPFKPKSVQNQDVIESTFSLFPETNLFIQGLSKIIQSLHWNQFVIIYEEEDNLIKLQDVLKLQQYKDGSDKNDMLIKQLGTTDDYRPLLKEVK
ESHITRIVLDCNPNKIIPILQQAKEVKLLHDISTSIFLSSLDAHTVDLSNINLGTNITTVRLFNPYNEGFQRIANQYYPAIPPEKIKVETALRHDSMILFLEVINSLS
SKNIIPQSTPINCTNLSSNIDDNHRIALINEMTNTTITNGLTGDEIHLEFNGKREVFHLELIEIDRPDRPIALWNSEVPDVVQFTRNATEREAELQRRLSSHNFIVS
SKIGKPYLMIDDENARGNARYKGYSMDLITEIAKIMNISFEFVLTANNLHKNIVEDLINRRADLGVCDFTITPQRSEVIDFSMPFMSLGISILHKESNDEETTN
MYAFMDPLSWRVWIYIVTLYLVLSLALLLIARLDPDDWENPHPCNQQPEELENIWGFRNCLWVTLGSIMTQGCDILPKGISSRIATAMWWFFSLILTSSYTAN
MTAFLTKQKSGDTINNVEELAKQNKVKYGFMKGGSTQDFFKFSNNSLYQKMWAVMKNDPSVFEDTNDRGVDRVLSTKNALYAFFMESTQIEYESERKCDL

KKIGQYLDSKSYGIGMPFGAEYRHSINAAVLRLQENGKLSELKTRWWKQNLDGIPPCPSSSDESANQDELTLSHVGGVFVVLAAGIGLAFLIAIIEFLLNVRN
ISIEEHMTYFEALKVELKFSSNIWITKKRVKPATSSSNSSSVKLDDTQSMARTILAGAGSVLNINASVLNRMAGYTR
>GluR5
MNKFSWIVITIVVTISGVCSSVKQDRLNIVIFLDEEQHDEVTLAAIKNAADIIKAKARYPVMSHIIEIRKDNTYEAGQIICGLLSKGIAAIFGPQSFEINEIIQSVS
QTLQIPHFQTFWDPKLQQLSPPSSSLSLSSTLFNLHPSVSDLSKALATLVRDNDWKSYTVIYENDDGLLRLRESLKQRRVTDLTMTFRKLGNGPDYRSVLKQI
KTSEQTHFILDCKAEKIFDVLRQAKEVKLLEDYHSYIFTDLDAHTLDWSQFPEMPANITAFRLIDPDSQAMRYVGKLWKTEPQTIKTSTALLYDALNVFFTSF
RDAEKHNEVEINTKMNCESLEEKFSSHGSQIVDAIRQPTSKHSRVILPGPLTGRITFDANGQRENFAIEIIETTKMKSEFRIAGKWNSKTPNSIYYTVTSEEREK
ELQKEIQQKNFKVVSRIGAPYLIPRKAEYGRTYYGNDRWEGYALDLMYEICNILNCSYTFELVPDGKYGNYDPVRKEWNGLIRHLLDRKADLAVCDLTITY
ERRIAVDFTMPFMTLGISVLYAKPIKEPPELLSFAHPLSLDVWLYTATSYLVISMIIFLVARLNPNDWENPHPCDQQPEELENIWGIRNCCWLTLGSIMTQGCDL
LPKGVSTRMATAAWWFFSLIMTTSYTANMAAFLTMSRMGLTIEKAEDLAGQSKIKYGCLAGGSTSSFFKDTNFSTYHQMWIQMESADPTVFETSNRDGVK
RVLTSKRKYAFLMESSSIEYETERNCDLIQVGGQIDSKGYGIAMTTNFQYRKAFNEAILKMQEMGILSRLKTKWWTEMNGGGQCNQEEESEDDAAAELGL
DNVGGVFVVLAAGVGIALIFATCEFLWMVKKVAVREHIGFKEAFKDELKFVLNIWARQKLVKKK
>GluR6
MALIKFLTTVTAVLVLFDYPKLVRSLPDIIPIGGLFHPTDDKQEIAFRYAVEKINSDRSILPRSKLQAQIEKIPPQDSFHASKKVCHLLRTGVAAIFGPQSPHTASH
VQSICDTMEIPHLETRWDYRLRRESCLVNLYPHPTTLSKAYVDLVKAWGWKSFTIIYENNEGLVRLQELLKAHGPYEFPITVRQLGEGIDYRPLLKQIKNSAE
SHIVLDCSTPRIYDVLKQAQQIGMMSDYHSYLITSLDLHGVDLEEFKYGGTNITAFRLVDPDGLEVRKAVRDWNFPPDSQKNKKSEIMTSFYRDNATFVKAE
TALMYDAVHLFAKALHDLDTSQQIDIKPLSCDAVDFWPHGYSLINYMKVVEMRGLTGVIKFDHQGFRTDFMLDIIDLTRDGLKKIGTWNSSEGVNFTRTYG
EAYTQIVEIIQNKTFVVSTILSSPYSMRKEASEKLTGNAQFEGYAVDLIHEISRTLGFNYTIKLAPDGRYGSLNRETKEWDGMMRELLDQKADLAIADLTITYD
REQAVDFTMPFMNLGISILYRKPIKQPPNLFSFLSPLSLDVWIYMATAYLGVSVLLFILARFTPYEWQNPHPCNPDPDHLENQFTLFNCMWFAIGSLMQQGCD
FLPKAVSTRMVAGMWWFFTLIMISSYTANLAAFLTVERMDSPIESAEDLAKQTKIKYGALRGGSTAAFFRDSNFSTYQRMWAFMESQRPSVFTSSNQEGVD
QVVKGKGGYAFLMESTSIEYVIERNCELTQVGGMLDSKGYGIAMPPNSPFRTAISGAILKLQEEGKLHILKTRWWKEKRGGGACRDDTTKTSSTANELGLA
NVGGVFVVLMGGMGVACVIAVCEFVWKSRKVAVEERESSLCSEMILELQHAFCCQPPQKTIKKPNDLSPNNKNERFNPTGSYSSYGFVASNNSIM
>GluR7
MFIKLIIVYYCLKVIHCDDTYNIVGFFEDDENNDVTLNGYVFDSAIAYTNEFKTPKFKIVGTNVTVIAQDPFKAITATCDSIREPIVAIFGPNSVNNIVAVQSVCD
SKEIPHILTRWMNTPLKLGSAINFYPHAAVLTEAYIDIIREWQWKTFTVLYENDESLLRLSGLILYAKEQGLVVTIEQLDRSDSQNYRESLKNVWRTKQRFLVI
DCSISILYEVLIQCQQVGLMTSDYNYLITNLDAHTIDLAPFMHSETNITGMRIINPQSNYVHQICEKLFPDEIAEKGVLQTAGELKTETALIFDAIQMFSDVLYR

MEQFQHPFGQPLDCLEPDSWKIGYSVVNSLKTSSYRGLTGLIEFNNEGNRSVFGLQIYELKEGGIITVAQWNSTEGINITRKHVKPPDLEKDSMRNKTFIVIIS
LTEPYGMNKETTETLYGNDRFEGFSIDLIQELAKLEGFNYTFVVQEDKKNGNIVNGRWTGMIGEVIEGRADMAITDLTITSDRAEAVDFTSPFMNLGVTILFQ
KATKTPPSFFSFAQPFALDTWIALAVAFVVVSLSFFLLGRICPDEWTNPYPCVEEPEYLLNQFSMTNAVWFATGAMLQQGSEIAPIAIPTRLVSGVWWFFVLIM
VSSYTANLASFLVTESHEELFNDVESLVANAEKHGISYGAKANGATIDFFEKSTENNTLHATIAKYMREHEMPNDNDKGVEKAERERFAFFMESTTIEYITQR
HCNLTMVGDRLDEKGYGIALKKDSPYRTRLSLAILKLQTSGEIEKIRKKWWEERKGGGQCTGATEDQTATPLDLQNVEGIFYVTIFGTILGVVLVIFEFLANIS
RISKRNQMPFKETFIREVKFFFKFNSNVKPLINANDDDDEDVEEEQNPIDIVSRQNSELRRSQTKSVKSKMTTNGSGPQYGYVVDRIGDRL
>GluR8
MNSFLWFNFIQFLFIINFGELKNLEYKVGGLFSERIHQVAFELIARELNNENSDTSFDISIEPFNVSYFNSLEAQIDVCTLFDRGVIGIFGPSSIYTSNHIQSICDQK
EIPQIEIHYDTKITRNKCVINLHPHHYEIISFFNHLILRFEWNRFIVLFEDNESLLRLEPLLELNMEYNVELIFRQLDKDASGSYREILNNIKKMPEKNILLDCSID
ILQDVLVQAQQVGLMTDDYSYIIMNLDFSIINLESFQYGGTNITGARVFNPEDEKTSKFHQSILEQLQDEQSLKMKLSTALLIDGMYLFHKVIQNLASAHEDI
LNSDVNCHSINSWNYGYTVANQIKSLVYENGITGRITFDVEGFRRDFKLDILELTENGLDKIGEWNSTAKSLNFVRAPDDDFQNDLTHQNIFNTTFKVITCLTA
PYAMLKETTDMLVGNDRFEGFCIDVIHELSLLLGFNYTFLIQEDGANGNLNRATNEWNGVIREIIDGRADFAITDLTITSERENAVDFTMPFMNLGISILYRKPE
PVPPSLFMFTSPFSTRVWIMLGVAYILVSIAIFIMGRLSPSEWTNPFPCIDEPEYLINQFSIRNSLWFTIGGLLQQGSELAPISISTRTASGFWWFFVLIMVSSYTAN
LAAFLTVETLVTPFKNIDELAKQTEIAYGAKSQGATQNFFRDSNVSSHKKVWQYLKTHPDVMTNDNDDGVLRVETKNYAYFMESTTIEYVVQRHCSLAQV
GGLLDDKGYGIAIKKYSPYRNDLSTAILKLQETGKLTQLKIKWWKEKRGGSTCGVKSEGGEAAALGLKNVGGVFLVLFIGALLALIGSFLEMAVGVYRKCK
LSKLSFKEEIKKEISFIIRFKANVKEITNQS
>GluR9
MDLILGNVLILSLLKICSTYGLPSFIKIGAIFMENQRGGAVEIAFRYAIYKINKDKMLLPHTSLIYDIQYVPDEDSFHASKKACRLVQNGVYAIFGPSDPLLAAHI
HSICDALDIPHLEARIDLESFTKEFSINLYPAQHLLNSAFENVINFLNWTKVAIIYEEDYGLIKLRELVRSPAHNRNLEIHLRQAAPEAYRDILKEIKTKEIHNIIV
DTKPSNMQLFLKAILQLQMNDYKYHYVFTSFDIETFDLEDFRYNFVNMTAFRLVDIDQLYVKEILRDMTRFQANLKLQPINSSFIDAQSALMYDSVFVFAIG
LQTLEQSRSVELTNVSCDSEQPWDGGLSLINYINAVEFKGLTGPIEFKEGKRIRFKLDLLKLKQHALMKVGEWYSDNGVNITDRNAFYDHGNMNVTLIVTTI
LEQPYVMLKSPSLNLTGNNRYEGFCLDLLKELATMIGFEYKVVLVPDGKYGAIDLETGEWNGIVKQLMDKKADLAVGSMTINYARESVIDFTKPFMNLGISI
LFKVPTDKESAFFTFLDPLGIKIWLSTMGAFFMAGFTICALGRFTPYEWVNPRPWQRNVKLVNQMNVSNSFWFITGTLLRQGSGVTPQATSTRIVGGIWWFF
TLIIISSYTANLAAFLTVERMITPIENAEDLAGQTEILYGTLESGSTMTFFRDSMIETYKKMWRFMENKKPSVFVPTYEEGIKKVLEGNYAFLMESTMLDYVV
QRDCNLTQIGGLLDSKGYGIATPMGSPWRDKISLAILELQEKGEIQMLYDKWWKNTGETCNRNEKGKENKANSLGVDNIGGVFVVLLCGLAFAVIVAIIEFC
YKFKKSALKKKRIQNAPHQSLCSEMVAELCFAMRCPGSRQKPALKRQCSKCLLGYSNTCSPIDILPFPPTPPPPPKQSQYKQNVP

>GluR10

METKKSAFVFLLHVVVVLLIFMHFQCIEGSLSEKIPLGAIFEQGTDDVQTAFKFAIANHNQNESGRRFELQAYLDVIKTSDAFKLSRLICNQFSRGVFSMIGAV
SPDSFDTLHSYSNTFQMPFITPWFPEKMQTLSTGSLDYAVSMRPEYHQAIVDTIRYYGWRRIIYLYDSNDGLLRLQQIYQALVPGNEYFQVKTVRRISNTSEA
LHFLKGLEEQSRWDIKYVVLDCSADMAKEIVIAHVRDIELGRRTYHYLLSGLVMDDRWESEVIEYGAINITGFRIVDSTRKNVRDFIEGWKRLDASQSFGAG
RETISAQAALMYDAVFVLVEAFNKILRKKPDNFKNINTGRTGRGQFYNNGTRMLDCNASGGWVTPWEHGDKISRYLRKVEIEGLTGDIRFNEDGHRQNYT
LHIVEMTVNSAMVKVAEWNDATGFSPVAAKYTRLQPQAVFERNKTYVVTTIVEEPYIIVRKEEPGEYLVGNDHFEGYCKDLADLIAKKLNINYELRIVKDG
KYGSENSDVKGGWDGMVGELVRNEADMAIAPITISSARERVIDFSKPFMSLGISIMIKKPMKQKPGVFSFLNPLSQEIWISVIFAFVGVSIVLFVVSRFSPYEW
RILHVTDEPVRHQPHLHMNSGGTMANDFSLLNSLWFSLAAFMQQGGDISPRSISGRIVGACWWFFTLIIISSYTANLAAFLTVERMVAPINSPEDLASQTEVE
YGTLMSGATWDFFKRSQITLYSRMWEFMNSRKHVFVSSYDEGIKRVRKSKGKYALLVESPKNDYTNERQPCDTMKVGRNFDAKGFGVATPLGSPLRDAVN
LAVLSLKEDGELTKLKNKWWYDRTECLKDKQESPRNELSLSNVAGVFYILIGGLFLAMGVATLEFCYKSHLEAKRAKIPISDAIKNKARLTLGVGRDCDNGK
RKKLEKKSSSQQQLNINNRYTSSPPLPQSKSATPQLTTKHNQTNTQQQQQQQYRCPHHFSSENGNYTLPRNSSQHQHQHLYYQPSYHNYEYNTSLNQQHKH
AHHQHQHSYQQEQPSTPTLNKI

>GluR11

MKLTIFALNLLVASVVSGGPAWKDIQRGTGLKVGEKGGRNTSASRGNGIKIGSSNRSSRITTSTTTVLPEEDHIYPPTSPSGGSNGQTMMTVGMIVPYKSFGT
RDYIKAVAQTKSLIGRKLKLFKTHDIDISVNMQPITPAPTSILKSLCKDFLNLNVSAILYIMNYEQYGRSTASTQYFFQLAGYLGIPVIAWNADNSGLERTASQS
SLQLQLAPSLEHQTAAMLSILERYKWHQFAVVTSLIAGHDDFIQAVRERVSAMQDRFKFTILNAILVANKGDLAALVDSEARVMLLYCTRDEAIDILTAAGDL
HLTGENYVWVVTQSVIQSTFQAPFQFPVGMLAVHFDTSSQSIVNEIMAAIKVFSYGVEDYLADPANRHRSLTTHLSCEGVEVSRWETGDYFFRYLRNVSVE
AEAGRPNLEFTPDGVLKAAELKILNLRPGGSKQTVWEEIGVWKSWQKEGLDIKDIVWPGNSHTPPQGVPEKFHLKITFLEEPPYIKLAPPDPITGKCSLDRG
VLCRVASDEQITEVDMAQAHRNGSYYQCCSGFCIDLLQKFSEELGFTYELVRVEDGKWGTNQNGKWNGLIADLVNRKTDMVLTSLTINAEREAVVDFSVPF
METGIAIVVAKRTGIISPTAFLEPFDAASWMLVGVVAIHASTFTIFLFEWLSPSGFNMKLSFNNGLSSTAHRFSLFRTYWLVWAVLFQAAVHVDSPRGFTSRFM
TNVWALFAVVFLAIYTANLAAFMITREEFFEFSGIDDHRLSRPFSHKPSIKFSTIPWSHTDSTLAKYFKEMHTYMRQFNKSTVLKGVAAVLSGDMDAFIYDGT
VLDYLTSQDEDCRLLTVGSWYAMTGYGLAFPRNSKYLKMFNKRLLDLRENGDLERLRRYWMTGACRPGKQEHKSSDPLALEQFLSAFLLLMAGILLAAL
LLLMEHLYFKYIRKHLAKTDRGGCCALISLSMGKSLTFRGAVYEAQDILRFHRCRDPICDTHLWKVKRELDMAQLRIKQLEKEMEVHGIKRPPCKRIVVSGE
QARARLRSLEQIDGGSCSDINGPTTTEIAEMETVL

>GluR12

MRPDEVLNILDSIEGNTVMEVEYSLPSYAASQNSLCVTSKITDVTLERVDGSLGITLRGGYVPEHPHLSRPLIITHIRPNGPAHRSSLIRVGDRLLKVDRHSLIN

KTLLEAQQILKESSNCNSHGITLTTLTIEYDVSVMESVKYANGPLLVEIDRQVEEDFGLILSNCNSMLNAQSAPDDILIAGFFIDRIVPGSTADRCGALCIGDQL
LAIDDLLLDTWTGSQGDAEKLLRRATKLQVLPFSVMQKAQSRSGFSGQFSGSSSSIAGFSTLNSKRSRNSRANRTTNNRQSTINKSFESDCSSNYCGCSSTMG
GVSHPETLTLTLTADRGLGYGLTVSVGDHTENRSADILITRISSDSPAYRSSCLQVGDRIVSVNHQSNLTLQEITSILEMGADLTGGRTITLTTEFDVADTIVPSSG
IFTVKLAKRGPGLGITTTASKTQPEEPFIISEIRRGSIAHRTGTLHAGDRLLAIDNRPLDHLSLESAFEILQTSSNEIVTLKVEKTETENSNLFLDSVVYTVELHR
YGGPLGITISGSEDSGDPIVLSRLTEGGLAEKTGALHVGDRILAINGEILDHRPLSEAIRLLQTSGDRVQLKIARNLKNDSALLEEPRCSYSSPGLMSVDSAIHS
WDSSNTGESQNENTSELKDIESVLSEPLSYQDQIHDHDKDLTLT

>GluR13

MKQKYKNVLYLILIIHSIKINGEKEKEWISNPSTFNIGGVLSSNESKALFKETIDHLNFDSSFVPKGVTYYNTAILMDSNPIRTALNVCKFLIAKQVYAVVVSHP
LTRDLSSAAVSYTSGFYHIPVIGISSRDSGFSDKNIHVSFLRTVPPYSHQADVWVEMLKHFNYKKVIFIHSSDTDGRALLGRFQTTSQSLEDDVEIKVQVESVI
EFETGLDNFREQLMEMKTAQSRVYLMYANKHDAKIIFRDAAALNMTDAGYAWIVTEQALEADNVPEGILGLRLVNATNEKAHIKDSIYVLASALRDMNQT
EEITAAPKDCDNTAQIWDTGRVLFNFIRRQVLINGETGKVAFDDQGDRINAEYEIINIQRKKKKVAVGRHFFNRETNKMNLIVDEKSILWPGRKNIKPEGFMIP
THLKVLTIEEKPFVYVRKIEGLGTCNEDEIPCPHFNETHEITAKYCCKGYCMDLLKELSKRVNFTYSLALSPDGQFGSYIIKNTSIGGKKEWTGLIGELVSEQA
DMIVAPLTINPERAEFIEFSKPFKYQGITILEKKPSRSSTLVSFLQPFSNTLWILVMVSVHVVALVLYLLDRFSPFGRFKLANTDGTEEDALNLSSAIWFAWGVL
LNSGIGEGTPRSFSARVLGMVWAGFAMIIVASYTANLAAFLVLERPKTKLTGINDARLRNTMENLTCATVKGSAVDMYFRRQVELSNMYRTMEANNYLTAE
MAIADVKAGNLMAFIWDSSLLEFEAAQDCELVTAGELFGRSGYGIGLQKGSPWGDDVTLKILDFHESGFMESLDNKWIFQGNMQQCEQFEKTPNTLGLKN
MAGVFILVGAGIIGGMGLIVIEMIYKKHQMKKQKRMELARHAADKWRGCVEKRKTLRASTGQQRRIKSNGVNEAVTISHVVDKFQRIGGPIGGPERAWPG
DTDIRQRRIDEASGQQPIPRYLPGYTWDVSHLVV

SNMP

>SNMP1

MKFYNKQLTIGTAVVAVFSLIMDLWLLDFLVRFGVRDQMSLRKRNQVREIFLKIPFPLYFKIYFFNVTNPQDVQNGAIPILQEIGPYYYNEFKERINIVDNKGE
DSLTYTPYDIFKFNQEMSGELKDTDYVTIIHPFIVGIVNYVMVNTPAYISIINKSIPNMLPTNADSLFLTAKVKDILFDGIEIDCTTNKFPTSAVCVQLQSQIPGLK
VKENNEKIFLFSLLGMRNATKSYRIKILRGILQIQDLGRLMEVNGKKEINLWRSQECNIFHGTDGWIFPPLLKSETGLESYSVDLCRNIKLKHINDTTLKKLNV
RIYEANLGDQMNESSEKCYCPLTGNCQKKGTFDVSKCMGVPIIATLPHFLHTDSSYLKLVNGLKPNEDKHSLRVYFEPITGTPIRAYKRMQFNFDLQNNQKIS
LFSNLSNCLFPLIWIEEESDLEGPLLKKLQLIFLFISITRYFFMTILLTSIMYLGFMLHQYRKNNRQIQISQEKEMENERKNMENDNTNYYEIKLRDNIKINKTN
NKVLSGHEFEKYQRY

>SNMP1a

MHSPAKLGIAGLITMVVTIIFGFLLFPEFIKTQIIKIVNLKPGNDIREMFLDVPFGLDFNIYIFSVQNPDAVQKGAKPELKEYGPFCYEEWKKKINISDNEDDDTI
TYQPVDTFYERHGLAGCADLNTEVTIPHPLILGMVNTVGRAKPGATSLLNKAIKSIFKNPTSLFITVKAKDLLFDGVSIPCGVKDFAGKAICTTLKNEPSIKHI
TEDELAFSFFWPKNGTAGKQMTALRGTKDFHDVGRIIKYDGASKMNVWNVRQCDEYRGTDGTIFPPFMKKKQGVVSFAPDLCRSLGATYGGKTEYNGISV
KIFTASLGDQSKNADEKCYCTTRDTCLKKGLMDLYKCLNMVVYVSLPHFYDSDESYLNGVKGLNPDPKKHGIKLLFELTTGSPLWARKRLQFNMPLEPNP
KVDVFNNFTSTVLPIFWVEEGVSLNKTMTKPLKTLFTMKKVVCICSALIVVGSLGALGTAGYIFMTTDKSLQFIKKDGTRVKPADEDLNIDTILGTDNAGYT
GEKF

>SNMP2a

MLFKKIKLNISYAKLYLLGIVSLIFLLITIYLGFMLLPDLIDKQIWKTKILYNNTEQWDNFIKTPFPFHFKVNFFNIVNPDEILMGTKPVVKEIGPYVYKLNRWK
DEIFWNKPTHISYYEYEKFEFDSENSKNLKENDIITTLNVPYASLLFKAESISIDFLMTLEPSLPIIFNNNLNQLFIKVKVKDLLFDGLRFCENNITNDDFTAKLIC
GQIKKEIKQYKNMRIDGDDILYSLLDYKNYKHQGYYTVNSGQSNNRDVAKLLLINNNSRITTWIDGGNSICNKIRGITTVFPNNLKESATFDTFSEDICRTIQM
TYSQTDLYENIVGFKFVANNLTFSNPEIDADNQCYCINKTMNLNKHFECFYNGILDLTNCKGAPIILSFPHLLYADSRYINTVEGLEANESQHETFVLLEPISGT
PLKLAKRFQFNMFIRSFEDVQIFENVTQSLIPIFWIDEGMNLPEHYINVLNNSLFRILHILNIVRWSILAISGFVLIFVLVSFLIHK
